# Description of a new and widely distributed species of *Bathypathes* (Cnidaria: Anthozoa: Antipatharia: Schizopathidae) previously misidentified as *Bathypathes alternata* Brook, 1889

**DOI:** 10.7717/peerj.12638

**Published:** 2022-02-08

**Authors:** Tina N. Molodtsova, Dennis M. Opresko, Daniel Wagner

**Affiliations:** 1 P.P. Shirshov Institute of Oceanology of Russian Academy of Sciences, Moscow, Russia; 2 U.S. National Museum of Natural History, Smithsonian Institution, Washington, DC, United States; 3 Conservation International, Center for Oceans, Arlington, VA, United States

**Keywords:** New species, Antipatharia, Seamount fauna, Deep-sea corals, Geographic distribution, Deep-sea mineral resources, Lower bathyal, Abyssal

## Abstract

For many years an undescribed species of the genus *Bathypathes* has been misidentified as *Bathypathes alternata* Brook, 1889 (a species currently re-assigned to the genus *Alternatipathes*). This new species is rather common at mid- and lower bathyal depths of the Pacific, Atlantic and Indian oceans, often in areas with high concentrations of commercially valuable cobalt-rich ferromanganese crusts, where it was observed in underwater photo and video transects to occur in high densities. Under the name *B. alternata* this species is recorded in several inventories and databases. There is an urgent need for a formal description of this misidentified and widely distributed species to avoid further confusion. The new species is superficially similar to *A. alternata* in having a monopodial corallum and simple, bilateral and alternately arranged pinnules. However, it differs from the former in that it has an upright corallum with a straight pinnulated part (*vs*. a horizontally bent pinnulated part), pinnules of uniform length and density (*vs*. decreasing regularly distally), and a constant distal angle formed by the pinnules and the stem along different parts of the corallum (*vs*. a decreasing distal angle near the top). The new species can therefore be easily distinguished from *A. alternata* in underwater imagery. We formally describe this new species in the genus *Bathypathes* and assign it the new name *B. pseudoalternata*. An extensive synonymy list with previous misidentified records is provided. To evaluate the distributional patterns of the new species we review the geographic distribution of antipatharians reported below 800 m. The majority of the hitherto described lower bathyal and abyssal species have been recorded from one biogeographic province; however, 20 species are known from more than two provinces, and only three species are widely distributed (>5 provinces), including the newly described *Bathypathes pseudoalternata*. Members of the family Schizopathidae, to which the new species belongs, represent the majority of the lower bathyal (50.54%) and abyssal (82.35%) species.

## Introduction

Corals, including black corals, are key components of hard-substrate ecosystems in the deep sea. Black corals are known to be an important habitat for a diverse range of organisms, providing food, shelter, nurseries and breeding grounds for number of organisms, thus enhancing biodiversity in the deep sea (Braga-Henriques et al., 2013; Wagner, Luck & Toonen, 2012; Yesson et al., 2017; De Clippele et al., 2019). Being slow-growing and expected to have slow rates of repopulation, black corals are primary indicator species for deep-sea vulnerable marine ecosystems including those associated with deep-sea mineral resources such as cobalt-rich crusts and polymetallic nodules (Molodtsova & Opresko, 2017). As a result, they are increasingly becoming the focus of conservation efforts (Braga-Henriques et al., 2013; Yesson et al., 2017). Therefore, it is important to be able to accurately identify the species at risk and to document the overall diversity of black corals in those areas. In addition, because information regarding the distribution and diversity of deep-sea organisms (*e.g*. cold-water corals) is increasingly being derived from imagery alone (Howell et al., 2019), there is a need to accurately identify species using gross morphological features whenever possible.

**Figure 1:**
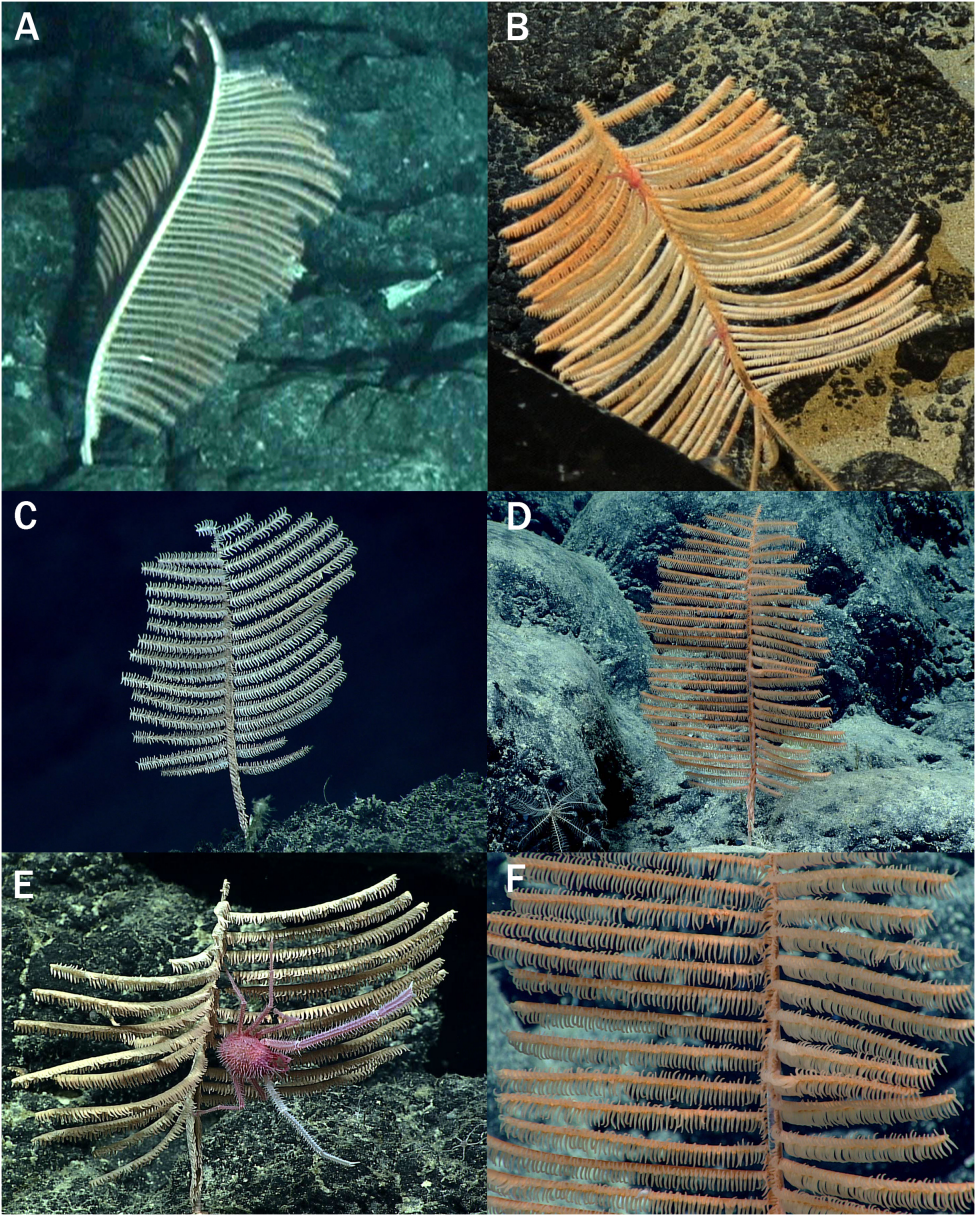
*Bathypathes pseudoalternata* sp. nov., *in situ* photos: (A) Holotype, USNM 1071044, *Pisces V*, Dive 534. Hawaii (B) Paratype, BPBM D1775, *Pisces IV*, Dive 228, specimen 12. Hawaii (C) NOAA Ship *Okeanos Explorer* EX1605 L3 Dive 15. Marianas Trench Marine National Monument (Cantwell, Pomponi & Fryer, 2019) (not collected). (D) NOAA Ship *Okeanos Explorer* EX2104 Dive 6. Corner Rise seamount chain (NW Atlantic) (Cantwell et al., 2021). (E) NOAA Ship *Okeanos Explorer* EX1703 Dive 7. Titov Seamount (Central Pacific) (Kennedy, Demopoulos & Auscavitch, 2017). (F) Same specimen as (D) close up showing associated polynoid polychaete. Photos courtesy of Hawaii Undersea Research Laboratory (A and B), NOAA OER (C–F).

For a number of years two species of antipatharian corals of the family Schizopathidae have been reported in the literature under the name *Bathypathes alternata* Brook, 1889, due to the superficially similar appearance of their corallum (Molodtsova & Opresko, 2017). Each of these species forms a monopodial corallum with simple, bilateral and alternately arranged pinnules. However, the holotype of *B. alternata* and conspecific specimens from the lower continental slope and abyssal plain of the North Pacific (2,670–5,089 m; Molodtsova & Opresko, 2017), demonstrate a very distinctive pinnulation pattern in which the pinnules show a regular decrease in length from the lower parts of the pinnulated section of the corallum to the apex. In addition, the distal angle that the pinnules form with the stem decreases from around 60° on the lower section of the stem, to 30° or less towards the apex of the corallum. Thus, the overall shape of the pinnulated section of the corallum is triangular. In this morphotype the stem of the corallum is often bent over such that it extends out almost horizontally to the substrate with the pinnules curved down towards the substrate giving the corallum a windsock-like appearance (Molodtsova & Opresko, 2017). In contrast, a number of specimens that have also been assigned to “*Bathypathes alternata*”, but reported from shallower depths (see *e.g*., Opresko, 1974; Wagner, Waller & Toonen, 2011; Barnich, Beuck & Freiwald, 2013; Brugler, Opresko & France, 2013; MacIsaac et al., 2013) have a very different colony shape and pinnulation pattern. These colonies are typically upright; the pinnules along most of the stem are similar in length, except those lowest on the stem and those near the apex where they are shorter and more or less straight (Fig. 1). Samples from the latter morphotype from Hawaii and the Northwest Atlantic have been sequenced using several different mitochondrial gene regions and the sequences have been deposited in GenBank under the name *Bathypathes alternata* (Brugler, Opresko & France, 2013; MacIsaac et al., 2013). These sequenced specimens were shown to have a close affiliation to species currently assigned to the genus *Bathypathes* Brook,1889, whereas a specimen having a similar pinnulation pattern as the typical form of *Bathypathes alternata* grouped with *Umbellapathes bipinnata* Opresko, 2005 (see Brugler, Opresko & France, 2013; Chery et al., 2018; Horowitz et al., 2020). *Bathypathes alternata* *sensu stricto* and *U. bipinnata* have since been assigned to the new genus *Alternatipathes* Molodtsova & Opresko, 2017. DNA sequencing studies indicate that specimens of the second morphotype, which have also been identified as “*Bathypathes alternata*” are not related to *Alternatipathes alternata* (Chery et al., 2018; Bilewitch & Tracey, 2020). Therefore, there is an urgent need for a formal description of this misidentified and widely distributed species to avoid further confusion, which is the purpose of this study.

## Materials and Methods

All examined material of the new species is listed in Table 1. Specimens evaluated in this study are deposited in: (1) the National Museum of Natural History (NMNH), Smithsonian Institution in Washington, DC, USA; (2) the Bernice P. Bishop Museum (BPBM) in Honolulu, HI, USA; (3) the National Institute of Water and Atmospheric Research (NIWA) in Wellington, New Zealand; (4) the Tasmanian Museum and Art Gallery (TMAG) in Hobart, Tasmania, Australia; (5) the Musée Océanographique de Monaco (MOM), Monaco; (6) the Muséum national d’Histoire naturelle (MNHN) in Paris, France; (7) the Discovery Collection of the National Oceanography Centre (NOC) in Southampton, UK; and (8) the P.P. Shirshov Institute of Oceanology (IORAS) in Moscow, Russia.

**Table 1:** *Bathypathes pseudoalternata* sp. nov. specimens examined as part of this study. Type material is highlighted in bold.

Sample Number	Locality	Latitude	Longitude	Depth (m)	Vessel or vehicle: Cruise	Station, dive or field number	Collection date	Remarks
**USNM 1071044** **(holotype)**	**Seamount southeast of Laysan Island, Hawaiian Islands**	**25.6689**	**−171.4110**	**1,195**	***DSRV Pisces V***	**Dive 534**	**20.10.2003**	**SEM stub 415**
**BPBM D1775** **(paratype)**	**off Nihoa, Hawaiian Islands**	**22.7395**	**−161.1724**	**1,327**	***DSRV Pisces IV***	**Dive 228 Spec. 12**	**03.10.2009**	**GenBank: KF054486, KF054613, KF054380**
USNM 96356	Kaulakahi Channel, Hawaiian Islands	21.9403	−159.8100	417–430	*RV Albatross*	Sta. 3998	14.06.1902	
USNM 98843	Penguin Bank, Hawaiian Islands	20.9897	−157.3190	418	*DSRV Pisces V*	Dive 300-Spec. PBS 05	19.09.1996	GenBank: AF052901
USNM 1071041	Pioneer Ridge, Hawaiian Islands	25.5742	−173.5060	1,742	*DSRV Pisces V*	Dive 526	09.10.2003	
USNM 1071043 (subsample USNM 1071412)	Seamount southeast of Laysan Island, Hawaiian Islands	25.7006	−171.4470	1,490	*DSRV Pisces V*	Dive 532	18.10.2003	
USNM 1163565	Necker Ridge, Hawaiian Islands	21.6343	−167.8210	1,748	*DSRV Pisces IV*	Dive 256-Spec. 6	14.10.2011	
USNM 1163568	Necker Ridge, Hawaiian Islands	21.6449	−167.8170	1,590	*DSRV Pisces IV*	Dive 256-Spec. 23	14.10.2011	
USNM 1163569	Central North Pacific	21.6428	−167.8250	1,500	*DSRV Pisces IV*	Dive 262-Spec. 12	19.10.2011	
USNM 1163570	Necker Ridge, Hawaiian Islands	21.5142	−167.9390	1,793	*DSRV Pisces IV*	Dive 257	15.10.2011	
USNM 1163571	Necker Ridge, Hawaiian Islands	21.5174	−167.9390	1,801	*DSRV Pisces IV*	Dive 257	15.10.2011	
MNHN IK-2012-12064	New Guinea	−6.4000	156.3330	1,045–1,207	*RV Alis*: SALOMON2	CP2232	29.10.2004	
IORAS CNI00015	Dobu Seamount, New Guinea	−9.7800	150.9717	980	*RV Akademik Mstislav Keldysh HOV MIR-2*	Sta. 2117	17.04.1990	
TMAG K1355	Huon Marine Park, Sister 1 (South) seamount mid	−44.2830	147.2670	1,364-919	*FRV Southern Surveyor*: SS199701	Sta. 14	23.01.1997	
YPM IZ 028567	Manning Seamount, North Atlantic	38.2188	−60.5120	1,340	*RV Atlantis:* AT08-01	Field number MAN204-1	14.07.2003	GenBank: JX560739, GQ200670, GQ200633
USNM 77110	East of Brunswick, Georgia	30.8700	−79.5700	658	*RV Oregon II*	Sta. 11717	21.01.1972	
USNM 83547	Yucatan Channel, Mexico	21.2833	−86.2167	412–457	*RV Pillsbury*	Sta. 587	14.03.1968	
USNM 1093063	off West Palm Beach, Florida	26.6516	−79.5424	759–776	*DSRV Johnson Sea Link*	Sta. 4915	11.11.2005	SEM stub 450
USNM 1139376	Stetson	31.8467	−77.6131	652–657	*DSRV Johnson Sea Link*	Sta. 4904	27.10.2005	
USNM 1139377	Savannah Banks	31.7411	−79.0974	519–543	*DSRV Johnson Sea Link*	Sta. 4900	22.10.2005	
Ifremer Brest reference collection (no number)	Bay of Biscay	47.7670	−12.3270	4,152	*RV Jean Charcot*: GEOMANCHE	CH60 DR13	03.03.1976	
MNHN IK-2012-12174	off Western Sahara	25.6500	−16.0330	822	*Talisman* 1883	Sta. 72	08.07.1883	
NOCS 9015	Continental slope of Morocco	28.7800	−12.3620	610–637	*RV Discovery*	Sta 9015 BN2-4	18.08.1976	
MNHN IK-2012-12009	Ampere Seamount	35.1000	−13.1170	2,010–2,100	*RV Le Noroit*: SEAMOUNT1	CP 102	12.10.1987	
MNHN IK-2012-12124	Bay of Biscay	44.0833	−4.3500	1,980	*RV Jean Charcot:* BIOGAS VI	CP 23	31.10.1974	
MOM INV-0021212	off San Miguel, Azores	38.0166	−25.3500	1,740	*l’Hirondelle II*	Sta. 3150	27.08.1911	
NIWA 4301	Hikurani Margin	−39.4300	178.4220	985–1,190	NZOI	Sta. R435	15.06.1990	
NIWA 4302	Three Kings Ridge	−31.1920	172.7900	1,100	NZOI	Sta. U606	10.02.1988	
NIWA 24195 (subsamples NIWA 4299; USNM 1174701)	Bay of Plenty	−37.1180	177.2840	690–800	NZOI	Sta. Z9225	15.08.1998	SEM stub 442
MNHN IK-2014-217	off Madagascar	−12.5950	48.2693	331–364	*FV Miriky*	CP3182	26.06.2009	
TMAG K4515	Great Australian Bight	−34.7977	131.7560	1,364	*REM Etive*: RE2017_C01	VSM02_101	18.03.2017	

Specimens were studied at IORAS and NMNH. At IORAS, microscopic skeletal features were examined using a TESCAN VEGA 3 LMU and Camscan S2 scanning electron microscope (SEM). At the NMNH, specimens were examined using an AMRAY 1810 or a Zeiss EVO MA 15 SEM. Fragments of pinnules up to 10 mm long were cleared of tissue, air-dried and coated with a 30–40 nm thick layer of 60% gold and 40% palladium prior to scanning. Measurements of the microscopic skeletal features were made using an optical dissecting microscope or light microscope equipped with an ocular micrometer or from the photographs taken under SEM. SEM stub numbers are from a specimen series deposited at the NMNH.

*In situ* images of colonies used for distributional records (Supplemental Material S1) were retrieved from the NOAA National Database for Deep-Sea Coral and Sponges Version 20210414-0 (https://deepseacoraldata.noaa.gov/) (Hourigan et al., 2015), the NOAA Benthic Deepwater Animal Identification Guide V3 (https://oceanexplorer.noaa.gov/okeanos/animal_guide/animal_guide.html), and Ocean Networks Canada SeaTube V3 (https://data.oceannetworks.ca/ExpeditionManagement). Only images of colonies that matched the external diagnostic features in overall colony morphology and branching pattern were included in the analysis. It is important to point out that diagnostic characters for this species in terms of polyp and spine morphology are not visible in *in situ* photos. Therefore, the species-level assignments of the photo records should be considered tentative.

The terminology used in the species descriptions generally follows that outlined in Opresko, Tracey & Mackay (2014). The size of the polyps, referred to as the transverse diameter, is the distance between the distal edge of distal lateral tentacles and the proximal edge of the proximal lateral tentacles of the same polyp. The polyp density is the number of polyps along a given segment of pinnule. The distance between spines is the distance between centers of the bases of adjacent spines in the same axial row. The height of a spine is the distance between the apex and the center of the base of a spine. The number of axial rows of spines is determined as the number of complete rows (those in which the base of the spines is visible) that can be counted in one lateral view (also referred to as one aspect). Counts and measurements for selected specimens of *Bathypathes pseudoalternata* sp. nov. are provided in Table 2.

**Table 2:** Counts and measurements for selected specimens of *Bathypathes pseudoalternata* sp.nov.

	Colony			Pinnules			Polyps	Spines	
Mus. No.	Height, cm	Stalk, cm	Pinnulated section, cm	Max length, cm	Density, 3 cm^−1^ P→D	TD, mm	Density, cm^−1^	Polypar/abpolypar [axis diameter], mm	Density, mm^−1^
NORTH PACIFIC OCEAN									
**USNM 1071044**	**24+**	**M**	**24**	**10**	**8→10**	**3.5**–**4.5**	**~2.5 [**	**0.04–0.045/0.02–0.03 [0.5]**	**5–6**
**BPBM D1775**	**42+**	**6+**	**37**	**15**	**5–6→8**	**3.5–4.2→5**	**3→1.5–2**	**0.038/0.025 [0.33]** **0.064/0.03 [0.38]** **0.036/0.026 [0.5]**	**~5**
USNM 1163565	25	5+	20	9+	6→5	2.8–4.5	3–2	0.03–0.04	NA
USNM 1163571	30.5+	M	30.5+	18	6→7	3–4	2–2.5	NA	NA
USNM 1163569	44	4	40	11	6→8	3.5–4.5	2–2.5	0.03–0.04	NA
USNM 1163566	30+	M	~30	13	7	3–4.5	2.5–2	0.03–0.04	NA
USNM 1071041	11	2.8	8.2	6.8	9	~4	~2.5	NA	NA
USNM 1071043*	NA	NA	NA	9	9	3–4	3–2.5	NA	NA
USNM 1071045*	NA	NA	NA	12	6–8	4–5	2	0.04/0.025 [0.3–0.5]	4**–**5
USNM 1163570	43	7	36	17	6→7	3–4	3–2.5	NA	NA
USNM 98843	22.5+	M	22.5+	~20	10→9	2→3	5→3.5	0.03–0.045	4**–**4.5
USNM 96356	67+	5	62+	16+	11–7.5*	2.8	3	0.06/0.04 [0.4] 0.07/0.04 [0.58]	NA
NORTH-WEST ATLANTIC OCEAN									
USNM 77110 (A)	29+	4	25+	~17	8	4–5	2.5 [4]	0.03–0.04 (E)	5–6
USNM 77110 (B)	16.8+	6	10+	9	8	3? (E)		0.03–0.04	5–6
USNM 83547	10	3	7	5.5	11–12	2.5–3?	3–4?	0.025–0.03	NA
USNM 1093063	45.5	5	41.5	15.5	9→7	~5	1.8–2	0.05/0.04 [0.36] 0.058/0.038 [0.55]	5
USNM 1139376	29+	4.6+	24.4+	~15.4	11	–	–	NA	NA
USNM 1139376-C	35+	6.3+	28.7	16	9→10	–	–	NA	NA
USNM 1139376-D	39+	3.9+	35	14	8→7	~4.5	–	NA	NA
USNM 1291076	16+	2+	14	4	10→12	2.5–4	3.5–2.5	0.03	NA
NORTH-EAST ATLANTIC OCEAN									
Ifremer (no number)	25	4	21	8.5	10→15	~4	2	0.04 [0.44]	5–5.5
MNHN IK-2012-12174	26+	4+	21+	17	7→9	2–4.2	2	0.04–0.06 [0.9]	5–5.5
MNHN IK-2012-12009	10	6.6	3.5	5.5	7→9	3.5–4	2	0.03–0.04 [0.3]	5–7
MNHN IK-2012-12124	6.5	3.5	3	NA	10	NA	NA	0.03–0.04 [0.3]	6–8
MOM INV-0021212	12	9,5	2.5	7	10→13	3.5→4	2	NA	NA
IORAS CNI00016	16.3	3	13.3	8	7–8	3–4	2–2.5	0.03–0.04 [0.3]	6–7
NOCS 9015	14.5	11	3.5	6.5	8→14	3.5–3.8	2–2.5	0.02–0.03 [0.37]	6–7
SOUTHWEST PACIFIC OCEAN									
MNHN IK-2012-12064	15.5	3.7	11.8	9	8→9	3–4.5	2.5–2	0.03–0.04 [0.3]	5–6
IORAS CNI00015*	NA	6	NA	13	7→10	5	2	0.05–007/0.04–0.05 [0.44]	5–7
NEW ZEALAND									
NIWA 24195	71+	1+	70	22	8–10	4	2.5	0.038/0.025 [0.7]	5–6
TASMANIA									
TMAG K1355	48+	2.5+	45.5	14	9→12	3–4.3	2.6–2	0.04–0.06 [0.65] 0.04–0.05 [0.56]	5–7
INDIAN OCEAN									
TMAG K4515*	NA	NA	NA	NA	5→6	5–6*	1.5	0.04–0.06 [0.45]	6
MNHN IK-2014-217	18.5	4.5	14	15	10→12	3.5–4	3.5–4	NA	NA

**Note:**

(*) Only fragment available for study. (+) Greater than. (D) Distal. (E) Estimation from photo. (M) Not collected. (NA) Not available. (P) Proximal. (TD) Transverse diameter. Type material is highlighted in bold.

We analyzed literature distribution records of deep-sea black corals (Supplemental Material S2), which were supplemented with unpublished distribution records validated by the authors (Supplemental Material S3). As fauna of the black corals in the mid- and upper bathyal zones is understudied, we restricted our analysis to black corals known from depths below 800 m, and included those in the lower bathyal zone (801–3,500 m) and abyssal zone (3,501–6,500 m; Watling et al., 2013). Currently accepted biogeographical provinces for these two depth zones (Watling et al., 2013) were used. To avoid ambiguities in the analysis of geographical distribution patterns, only species determined to species level or provisionally identified as new species were considered (Supplemental Material S2).

The electronic version of this article in Portable Document Format (PDF) will represent a published work according to the International Commission on Zoological Nomenclature (ICZN), and hence the new names contained in the electronic version are effectively published under that Code from the electronic edition alone. This published work and the nomenclatural acts it contains have been registered in ZooBank, the online registration system for the ICZN. The ZooBank LSIDs (Life Science Identifiers) can be resolved and the associated information viewed through any standard web browser by appending the LSID to the prefix http://zoobank.org/. The LSID for this publication is: [urn:lsid:zoobank.org:pub:58FF4531-1478-4462-84ED-A7E96920710E]. The online version of this work is archived and available from the following digital repositories: PeerJ, PubMed Central and CLOCKSS. The holotype of the new species is deposited in the collections of the NMNH and the paratype is deposited in the collections of the BPBM.

## Results

**Systematic description**

Family Schizopathidae Brook, 1889

Genus *Bathypathes* Brook, 1889

partim *Bathypathes* Brook, 1889: 151

*Bathypathes*, Opresko & Molodtsova, 2021: 405–407 (see synonymy list therein).

**Diagnosis.** Corallum monopodial, unbranched or rarely branched, and pinnulate. Pinnules simple, arranged alternately or suboppositely in two anterolateral or lateral rows. Length of pinnules on stem and branches usually longest near the middle of the pinnulated section of the corallum. Striatum present or absent. Spines conical, smooth, usually simple, but in some cases forked or multiply knobbed at apex, with acute to slightly rounded apex. Spines often larger on polypar side of axis than on abpolypar side. Polyps elongated in the direction of the skeletal axis from 2 mm to as much as 17 mm in transverse diameter.

**Species assigned to the genus.** *Bathypathes alaskensis* Opresko & Molodtsova, 2021; *B. bayeri* Opresko, 2001; *B. bifida* Thompson, 1905; *Schizopathes conferta* Brook, 1889; *B. erotema* Schultze, 1903; *B. galathea* Pasternak, 1977; *B. patula* Brook, 1889; *B. platycaulus* Totton, 1923; *B. patula plenispina* Brook, 1889; *B. pseudoalternata* sp. nov.; *B. ptiloides* Opresko & Molodtsova, 2021; *B. tenuis* Brook, 1889; *B. tiburonae* Opresko & Molodtsova, 2021; and *Stichopathes robusta* Gravier, 1921.

**Remarks.** Although originally assigned to the genus *Bathypathes*, *B. alternata* Brook, 1889, has recently been reassigned to *Alternatipathes* Molodtsova & Opresko, 2017, and *B. lyra* Brook, 1889 was made the type species of the genus *Abyssopathes* Opresko, 2002. *Bathypathes tenuis* was originally described and figured as having branched pinnules suggesting an affiliation with the genus *Umbellapathes* Opresko, 2005. However, subsequent examination of the type material (UKNHM 90.4.9.24) revealed the presence of a striatum and the absence of secondary pinnules, suggesting an affiliation with the genus *Bathypathes* (Opresko & Molodtsova, 2021). The type material of *B*. *tenuis* is a very young specimen that was dried out and subsequently rehydrated, and is now in very poor condition. Thus, this species is considered *incertae sedis* (Opresko & Molodtsova, 2021). *Stichopathes robusta* Gravier, 1921, undoubtedly belongs to the family Schizopathidae and was thus recently re-assigned to the genus *Bathypathes* (Molodtsova, 2014). However, the type of *S. robusta* consists only of a single pinnule and nothing is known about the arrangement or density of pinnules, so we prefer to consider it here as Schizopathidae *incertae sedis*.

***Bathypathes pseudoalternata* sp. nov.**

LSID: [urn:lsid:zoobank.org:act:DE0DC39A-4A11-4AC1-9EB1-F632FE9E3D1F]

(Figs. 1–7)

**Figure 2:**
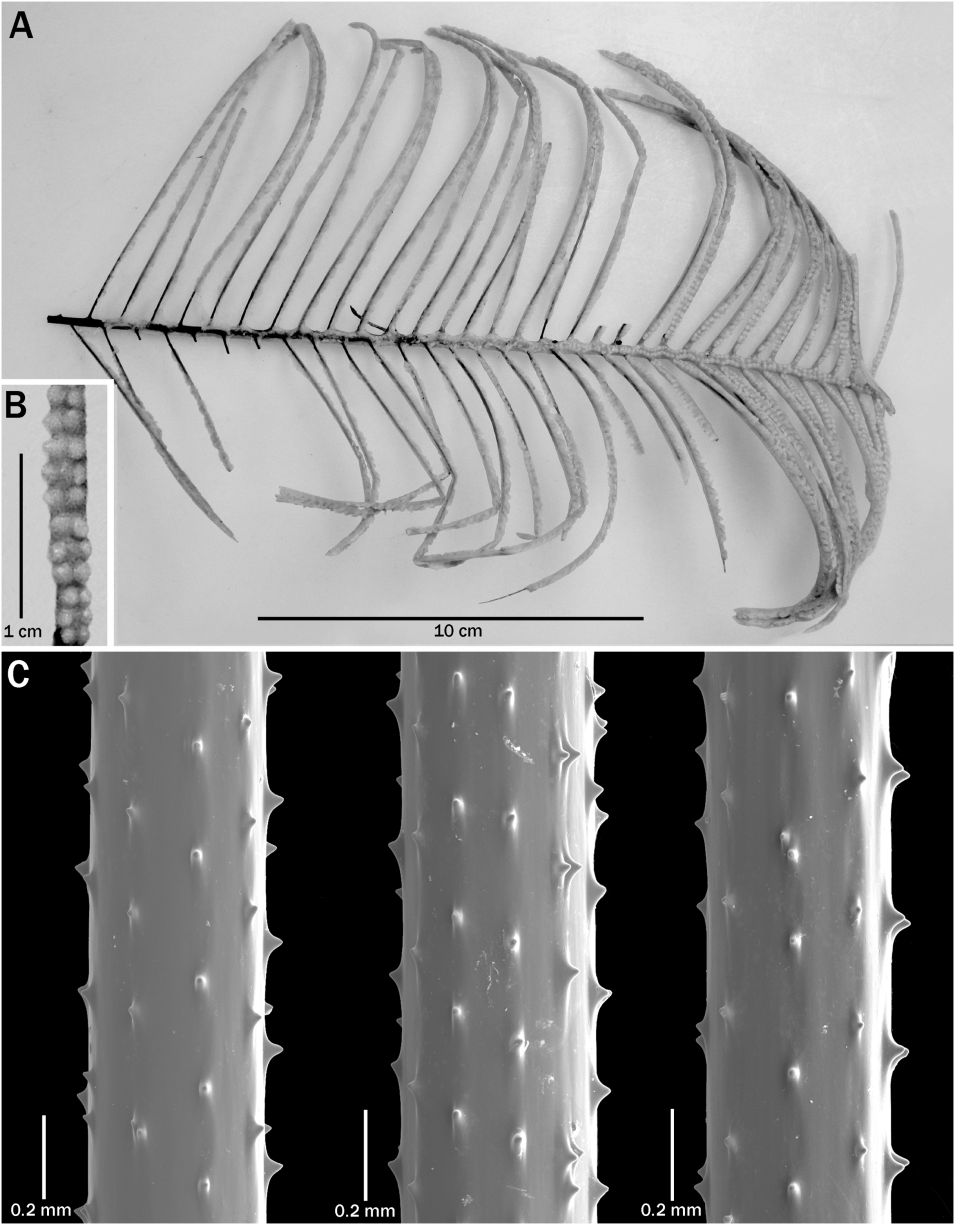
*Bathypathes pseudoalternata* sp. nov., holotype, USNM 1071044 (Hawaii). (A) Corallum. (B) Preserved polyps. (C) Sections of pinnules showing size and arrangement of spines (C from SEM stub 442).

**Figure 3:**
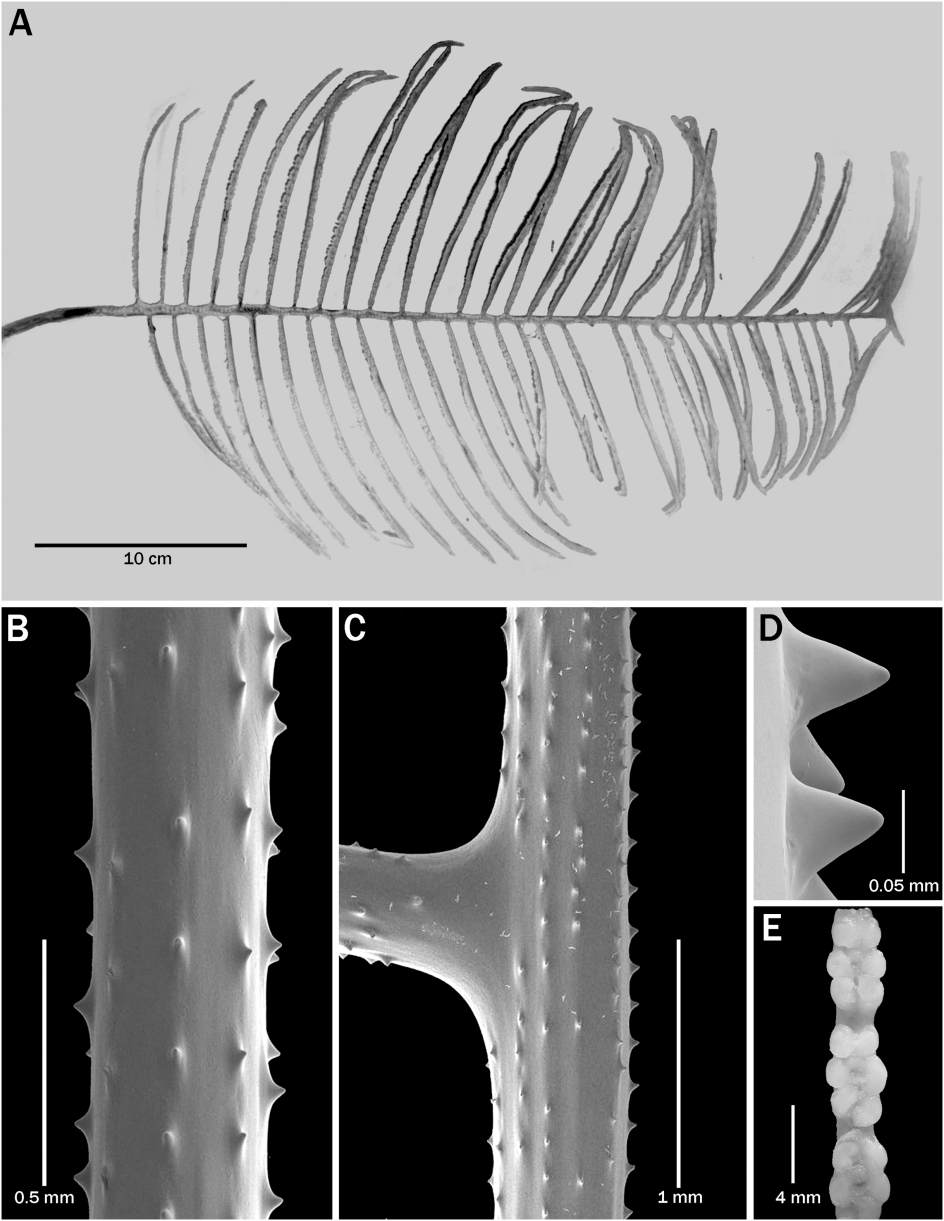
*Bathypathes pseudoalternata* sp. nov., paratype, BPBM D1775 (Hawaii). (A) Corallum. (B) Section of pinnule. (C) View of stem and base of pinnule. (D) Closeup view of spines. (E) Polyps.

**Figure 4:**
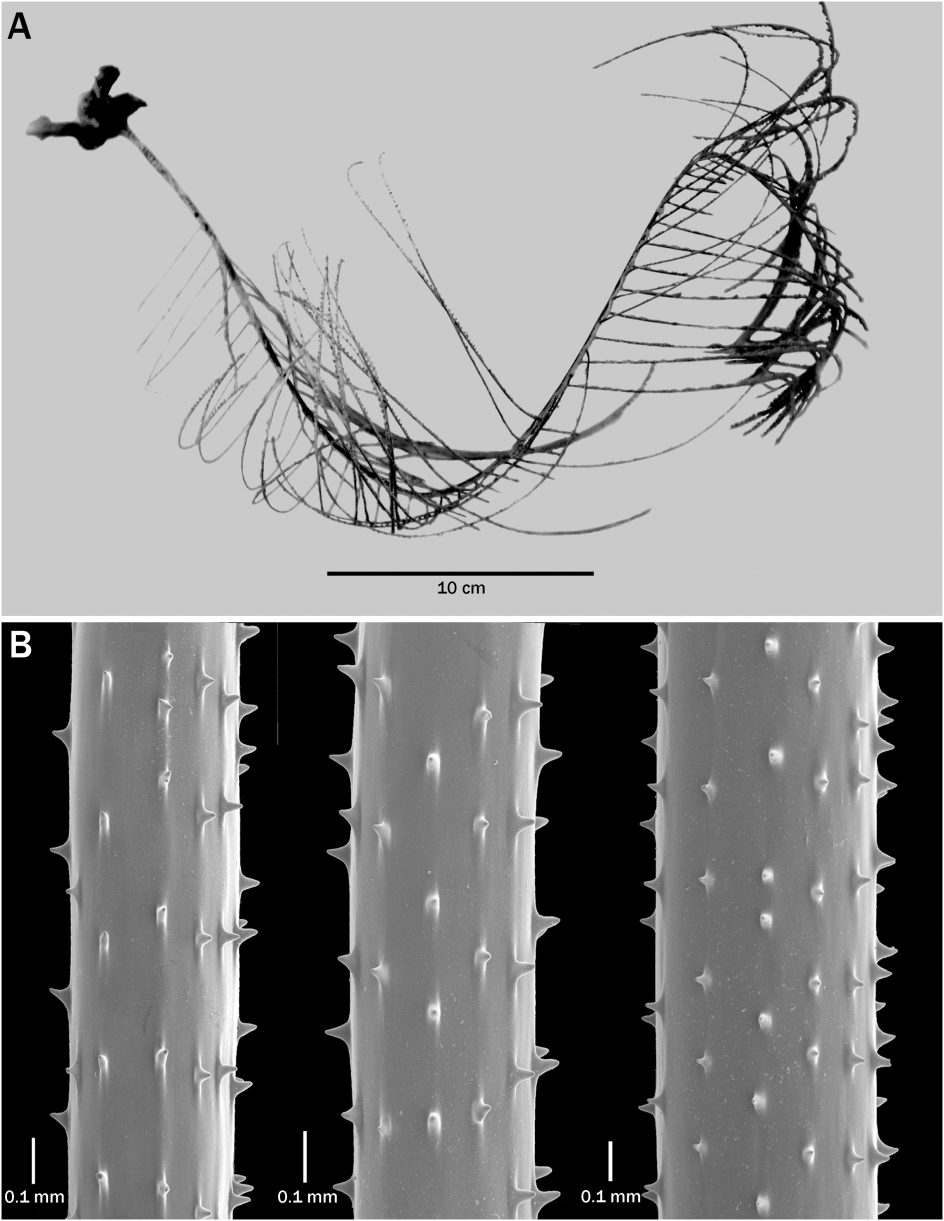
*Bathypathes pseudoalternata*, sp. nov., USNM 1093063 (NW Atlantic). (A) Corallum. (B) Sections of pinnules with spines (B from SEM stub 421).

**Figure 5:**
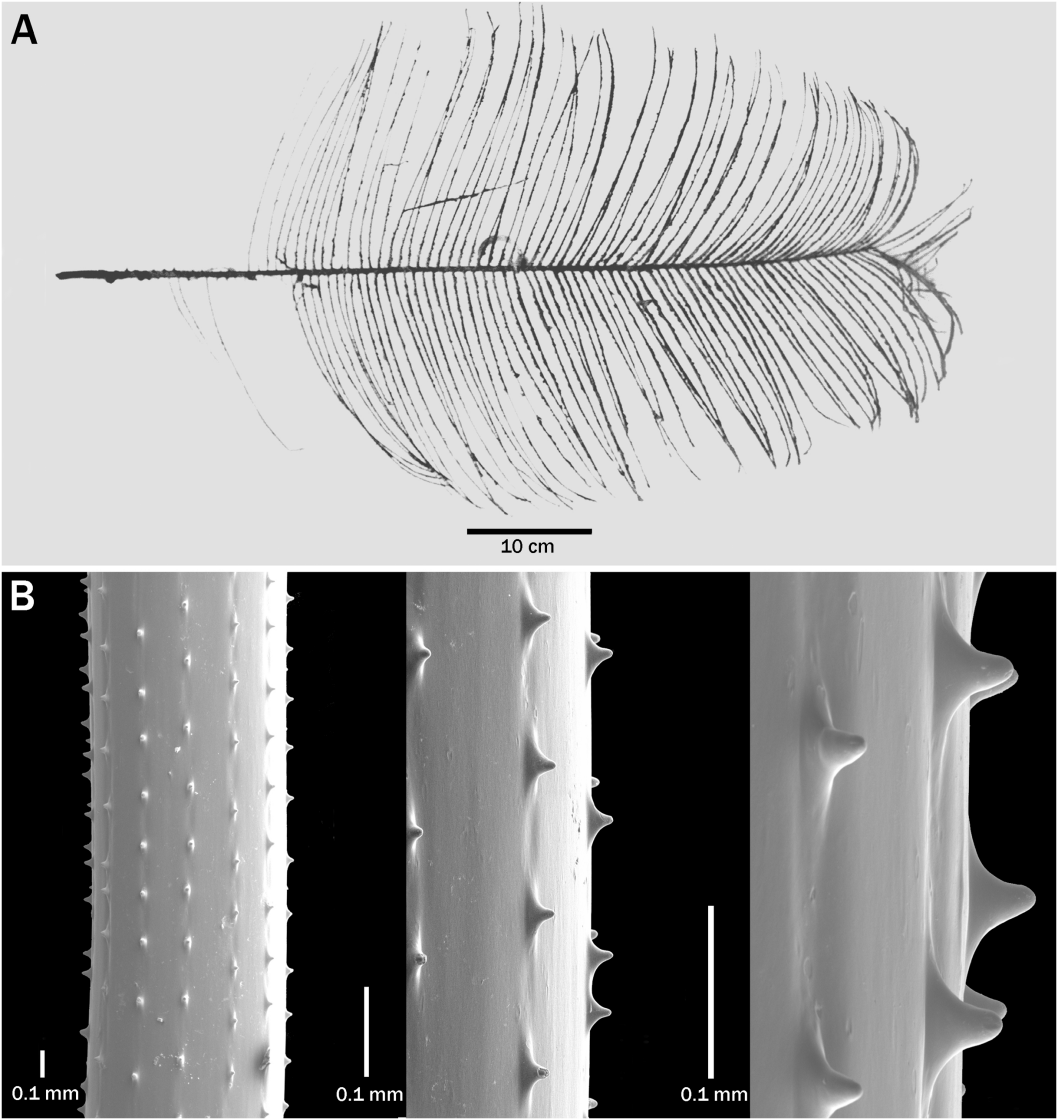
*Bathypathes pseudoalternata* sp. nov., NIWA 24195 (and subsample NIWA 4299) (New Zealand). (A) Corallum. (B) Section of pinnules showing spines (B from subsample USNM 1174701, SEM stub 442).

**Figure 6:**
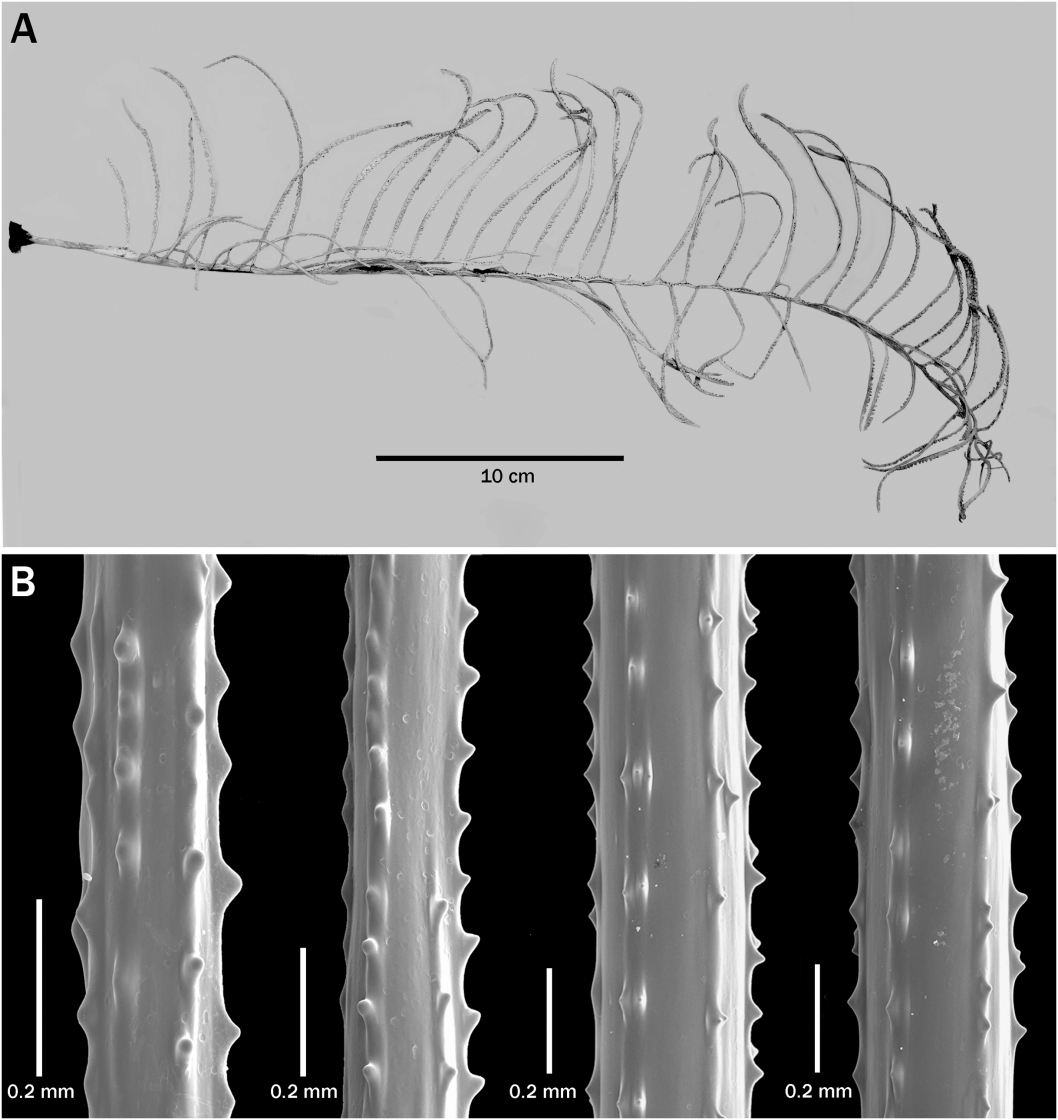
*Bathypathes pseudoalternata* sp. nov., USNM 1163569 (Hawaii). (A) Corallum. (B) Section of pinnules showing size and arrangement of spines (B from SEM stub 421).

**Figure 7:**
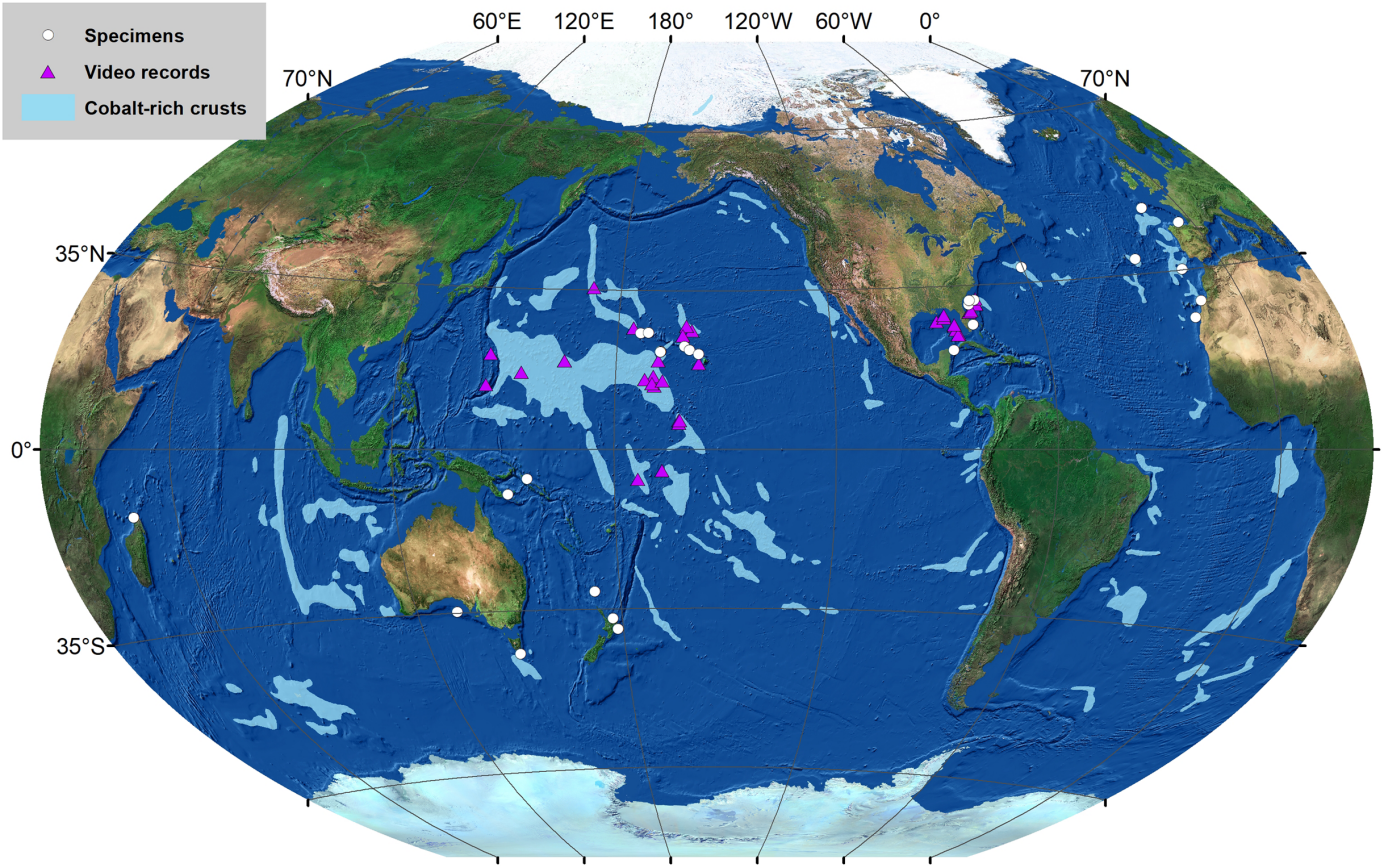
Map based on physical specimens examined as part of this study (Table 1), as well as photo records of specimens that show diagnostic characters for this species (Supplemental Material S1).

*Bathypathes patula*, Gravier, 1921:16 (in part); Opresko, 1974: 127 (in part); Bilewitch & Tracey, 2020: 1.

non *Bathypathes patula* Brook, 1889:151; Bilewitch & Tracey, 2020:12–13, fig. 2A, 2B.

*Bathypathes alternata*, Opresko, 2009:363; Clark et al., 2011:27, fig.11; Wagner, Waller & Toonen, 2011:215, 218–219, fig. 1(i), fig. 3; Brugler, Opresko & France, 2013:343, figs 2–3, 5, Suppl. Table S1; MacIsaac et al., 2013:240, 252–253, fig. 9; Bilewitch & Tracey, 2020:fig. 2A, 2B; Horowitz et al., 2020:559, fig. 3a.

*Bathypathes* cf. *alternata*, Barnich, Beuck & Freiwald, 2013:3 (specimen not studied); Molodtsova, Britayev & Martin, 2016:391.

non *Bathypathes alternata* Brook, 1889:153.

*Bathypathes robusta*, Molodtsova, 2014:5 (in part).

? *Stichopathes robusta* Gravier, 1921:12–13, Pl I(3–5), Pl. II(16–17).

*Bathypathes* sp. Cairns et al., 1993:5; Berntson, France & Mullineaux, 1999:423; MacIntosh et al., 2018:7; Etnoyer, Shuler & Cairns, 2020:3, 10; Hourigan et al., 2020:2; Lapointe et al., 2020:3–11 (in part), Suppl. fig. S8 (B).

*Bathypathes* sp. 1, Morgan et al., 2015:98, fig. 8.

*Alternatipathes alternata*, Parrish et al., 2020:3 (in part).

non *Alternatipathes alternata*, Molodtsova & Opresko, 2017:350, 358–360, fig. 6.

**Material studied.**

**Holotype.** USNM 1071044, seamount southeast of Laysan Island, Hawaiian Islands, 25.6689° N, 171.411° W, *DSRV Pisces V*, Dive 534, 1,195 m, coll. A. Baco-Taylor, 20.10.2003 (SEM stub 415).

**Paratype.** BPBM D1775, off Nihoa, Hawaii, 22.7395° N, 161.1724° W, *DSRV Pisces IV*, Dive 228, Spec. 12, 1,327 m, coll. C. Kelley, 03.10.2009 [GenBank: KF054486 (*IgrW*); KF054613 (*IgrN*); KF054380 (*cox3-cox1*)]. Note: the Genbank sequences were entered under the species name *Bathypathes alternata* and this name needs to be changed to *B. pseudoalternata*. The identity of other specimens in GenBank listed under *B. alternata* and *Alternatipathes alternata* needs to be verified to be certain they are not *B. pseudoalternata*.

**Other material examined.** See Table 1.

**Diagnosis.** Colony monopodial, unbranched, pinnulate. Lower unpinnulated section of stem relatively short, usually less than 7 cm. Striatum can be present or absent; when present it begins near the middle of the unpinnulated portion of the stem and generally extends past the second pinnule from the bottom. The striatum is generally more distinct on the abpolypar side of the stem. Pinnules simple, subequal in length over most of corallum, slightly shorter at the bottom of the pinnulated section and also decreasing in length near the apex; length usually about 10 cm in colonies approximately 20 cm tall and up to 22 cm in colonies 40 cm or taller; arranged alternately in two lateral to anterolateral rows along the stem. Spacing of pinnules slightly variable within colonies (increasing or decreasing distally along stem), but very variable between colonies (5–12 mm apart in each row); resulting in pinnular densities ranging from 6–8 to 10–12 (total per 3 cm) between colonies (or 8–18 pinnules total per 5 cm). Spines small, smooth, conical, with rounded apex; usually 0.03–0.05 mm tall (maximum about 0.08 mm on thicker pinnules); 4–5 per mm in each row, with 5–7 rows visible in lateral view. Double and triple spines may be present. Polyps usually 4–5 mm in transverse diameter (range 3–5 mm), resulting mostly in 2–2.5 polyps per cm.

**Description of holotype.** The corallum of the holotype (USNM 1071044) is about 24 cm tall and 15 cm wide (Fig. 2A). The entire unpinnulated lower section of the stem and holdfast are missing. Comparison of the *in situ* photo (Fig. 1A) with that of the collected specimen suggests that the lower unpinnulated section of the stem was about 6 cm long prior to collection. The diameter of the stem at the broken basal end is 1.9 mm. The pinnules are simple, bilateral to slightly anterolateral, strictly alternating, and similar in length along most of the stem. The longest pinnules are about 10 cm long and 0.6 mm in diameter near the base; they occur slightly above the middle of the pinnulated section of the corallum.

The two rows of pinnules form a wide interior angle, and the individual pinnules are directed distally. The distal angle of the pinnules with the stem ranges from about 60 to 70°. The pinnules in each row are about 8 mm apart proximally, decreasing to 6 mm distally along the stem, resulting in a pinnular density of 8 (total for both rows) per 3 cm on the lower part of the stem, but increasing to 10 per 3 cm distally (13 per 5 cm increasing to 16 or more per 5 cm distally). Along 10 cm of stem there are a total of 28 pinnules.

The pinnular spines (Fig. 2C) are small, conical, slightly compressed laterally, with a rounded apex, and flared out at the base in the proximal and distal direction. They stand out at a right angle to the axis. On a section of pinnule about 0.5 mm in diameter, the polypar spines are 0.04–0.045 mm tall and the abpolypar spines 0.02–0.03 mm tall. Five to six rows of spines are visible in lateral view. The spines are 0.15–0.38 mm apart in each row, with about 5–6 spines per mm.

The polyps (Fig. 2B) on the pinnules are mostly 4.0–4.5 mm in transverse diameter. The smallest polyps (~3.5 mm) are often found near the base of the pinnules. The interpolypar space is small, and the polyp density over most of the pinnules is typically about 2.5 per cm.

**Description of paratype.** The paratype (BPBM D1775, Fig. 3A) is about 42 cm high and 30 cm wide, with pinnules up to about 15 cm long. The longest pinnules occur about 25 cm below the top of the corallum and about 10 cm above the lowermost pinnules which are 10–11 cm long. The pinnulated section is 37 cm long; the unpinnulated stalk is 6 cm (the basal plate is missing); and the diameter of the stem at its basal end is about 4 mm. The alternately arranged pinnules are up to 12 mm apart proximally, decreasing to 7 mm distally resulting in a pinnular density of 5–6 per 3 cm on the lower part of the stem and increasing to 8 per 3 cm distally.

In the *in situ* photo of the colony (Fig. 1B), the interior angle formed by the two rows is about 120° on the lower part of the corallum, decreasing to about 90° higher up. In the preserved specimen, however, the interior angle is closer to 180° over most of the corallum. The distal angle of the pinnules with the stem ranges from about 75° to almost 90°. Pinnules on the lower parts of the corallum tend to be less inclined distally than those in the upper part of the corallum.

On the pinnules the polypar spines are up to 0.064 mm tall, and the abpolypar spines are up to 0.03 mm tall. There are 6–7 rows of spines. The spine density is 5 per mm on the pinnules and 8 per mm on the stem.

In the preserved specimen the polyps over most of the proximal section of the pinnules are 3.5 to 4.2 mm in transverse diameter; in the midsection and distally they are up to 5 mm in transverse diameter (Fig. 3E). The polyp density ranges from about 3 per cm lower on the pinnules to 1.5–2 per cm near the tip of the pinnules.

**Additional material.** We include here detailed descriptions of specimens from the western Atlantic and New Zealand, two localities very distant from the type locality, to illustrate the fact that based on our present information it appears that the species is cosmopolitan in distribution. The preserved specimen from the western Atlantic (USNM 1093063, Fig. 4A) has a 45.5 cm long curved stem which at its basal end is 2.7 mm in diameter. A striatum is present and starts at 1 cm above the basal end of the stem and extends for 5 cm beyond the point where the pinnules first appear. The lower 5 cm of the stem lacks pinnules. The pinnules in each row are spaced 7 mm apart on the lower part of the stem, increasing to 10 mm on the upper part. The pinnular density is 9 (total) per 3 cm on the lower parts of the corallum and 7 per 3 cm on the upper parts. The longest pinnules occur near the middle of the corallum and are 15.5 cm and have a basal diameter of about 0.6 mm. The interior angle formed by the two rows varies over different parts of the stem, from greater than 90° on the lower parts of the stem to less than 90° in the upper parts and greater than 90° near the apex. The pinnules are directed distally, with the distal angle being about 60°. The polypar spines (Fig. 4B) are 0.05–0.058 mm tall and the abpolypar spines 0.038 to about 0.05 mm. Seven or eight rows of spines are visible in lateral view and the spine density is 5 per mm. The polyps are close to 5 mm in transverse diameter, and there are 2 polyps per cm.

The specimen from New Zealand (NIWA 24195, Fig. 5A) is a 71 cm tall, upright colony about 40 cm wide, and with stem diameter 1 cm below the pinnulated section of 5 mm by 7 mm. The pinnules are up to 22 cm in length in the middle section of corallum and about 2 mm in diameter near the base. The pinnular density is 8–10 (total for both rows) per 3 cm. The polypar spines on a pinnule 0.7 mm in diameter are 0.036 mm tall and the abpolypar spines 0.025 mm tall (Fig. 5B). The small conical spines have a very rounded apex, and are arranged in axial rows with six to eight rows visible in one view on a section of pinnule 0.7 mm in diameter. Within each row the spines are spaced 0.16–0.26 mm apart, resulting in 5–6 per mm in each row. The polyps are about 4 mm in transverse diameter, with about 2.5 polyps per cm.

**Intraspecific variation**. The taxonomic characters of the specimens assigned to this species are shown in Table 2. They are divided into groups by geographic region. The data presented leads to the following conclusions: (1) colonies can reach 70 cm or more in height and are usually very upright; (2) the lower unpinnulated section of the stem is very short, less than 7 cm; (3) a striatum can be present or absent (if visible, it begins in the middle of the unpinnulated section, normally 1.5–2.0 cm below the first pinnule and continues up to the base of fourth to eleventh pinnule); (4) the maximum length of the pinnules in the largest colonies is about 20 cm; (5) the pinnules are subequal in length over most of a colony with the exception of those lowermost on the stem and the newly developing ones at the apex; (6) the length of the pinnules is not necessarily correlated with the size of the colony; (7) the spacing of the pinnules within a row ranges from 4–12 mm, but is most often 6–10 mm; (8) pinnular density (total for both rows) ranges from 8 to 18 per 5 cm between colonies, but varies little within colonies or increases or decreases slightly distally; (9) the interior angle formed by the two rows of pinnules is very variable and ranges from less than 90° to 180°; (10) the distal angle of the pinnules is usually 60–80°; (11) polypar spines are typically 0.03–0.04 mm tall, but can be up to 0.07 mm in some colonies and on the thicker pinnules; (12) the spines can split to form double spines and additional rows; (13) the transverse diameter of the polyps is generally 4.0–4.5 mm, but can range from about 3 to 5 mm, with the smaller polyps often found at the proximal end of the pinnules; and (14) the polyp density is most commonly about 2.5 per cm, but can range from of 2 to 3 per cm. In the largest specimen examined (USNM 96356, >67+ cm) a striatum is not visible.

There are slight differences in the morphology of the spines in the examined specimens. In the specimens from Hawaii, the spines are generally triangular, with a wide base that extends out along the axis in both the proximal and distal direction (Figs 2B and 3B–3D), whereas some colonies from the Atlantic have spines that are narrower, more upright and a bit more conical (Fig. 4B). Some specimens from New Zealand have spines with a strongly rounded apex (Fig. 5B).

One of the Hawaiian specimens assigned to this species (USNM 1163569, Fig. 6A), differs from the typical form in that the spines tend to lie along very distinct ridges (Fig. 6B), a characteristic that also occurs to varying degrees, in other Hawaiian colonies. This specimen also has relatively short pinnules given the size of the colony. The pinnules are not more than about 10 cm long, although the entire colony is 44 cm tall, more than twice the size of the holotype which has pinnules of about the same size.

**Associated fauna.** A specimen from the eastern Gulf of Mexico was reported to harbor the polychaete *Eunoe purpurea* Treadwell, 1936 (Polynoidae) along the main stem (Barnich, Beuck & Freiwald, 2013: fig. 4a). In a similar way many colonies from the North Pacific, including smaller colonies 16 cm high were observed to harbor similar unidentified polynoid polychaetes (identification T. Britayev and D. Martin; see Figs. 1B and 1F, Supplemental Material S4). The polychaete was always observed nestling along the polyps on the main stem of the coral, with the tentacles forming a soft tunnel around the polychaete worm. Neither worm-runs nor apparent changes in pinnule arrangement, as often described in other associations of scale-worms with antipatharians (Molodtsova, Britayev & Martin, 2016), were reported. Several species of squat lobsters of the family Chirostylidae were also reported in association with *B. pseudoalternata* sp. nov. (determination from photographs by E. Macpherson; see Fig. 1E, Supplemental Material S4), however, there is no indication of species-specific associations.

**Figure 8:**
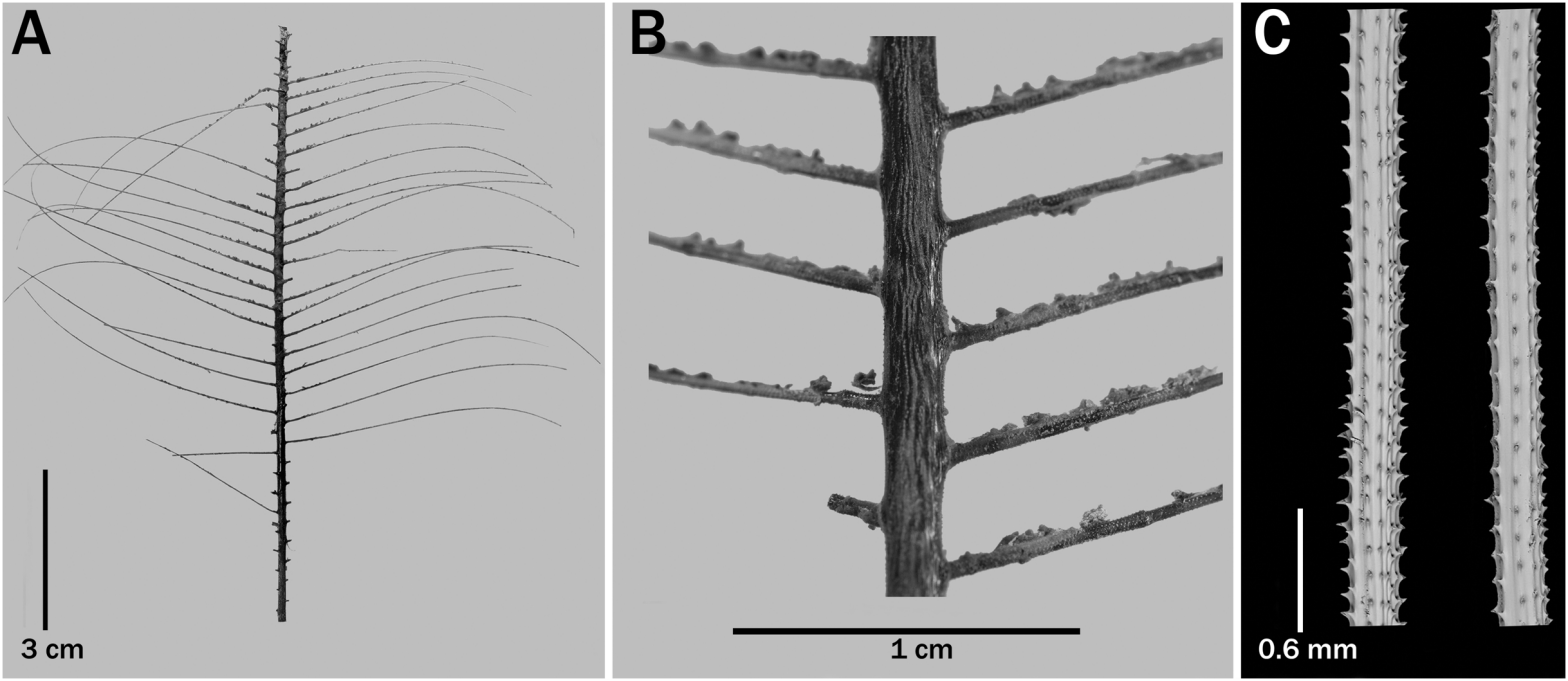
*Bathypathes platycaulus* Totton, 1923, holotype, UKNHM 1923.19.19.2. (A) Corallum. (B) Section of stem from abpolypar side. (C) Pinnules.

**Comparisons.** Most of the currently known species in the genus *Bathypathes* have pinnules arranged in subopposite pairs. The only other species of *Bathypathes* with alternately arranged pinnules is *B. platycaulus* Totton, 1923 (Fig. 8). It also forms an upright bilaterally pinnulate colony; however, it differs from *B. pseudoalternata* sp. nov. in having much more densely arranged pinnules (25–30 per 5 cm *vs*. 8 to 18 per 5 cm), thinner pinnules and smaller polyps (mostly 2–3 mm in transverse diameter *vs*. 3 to 5 mm).

**Figure 9:**
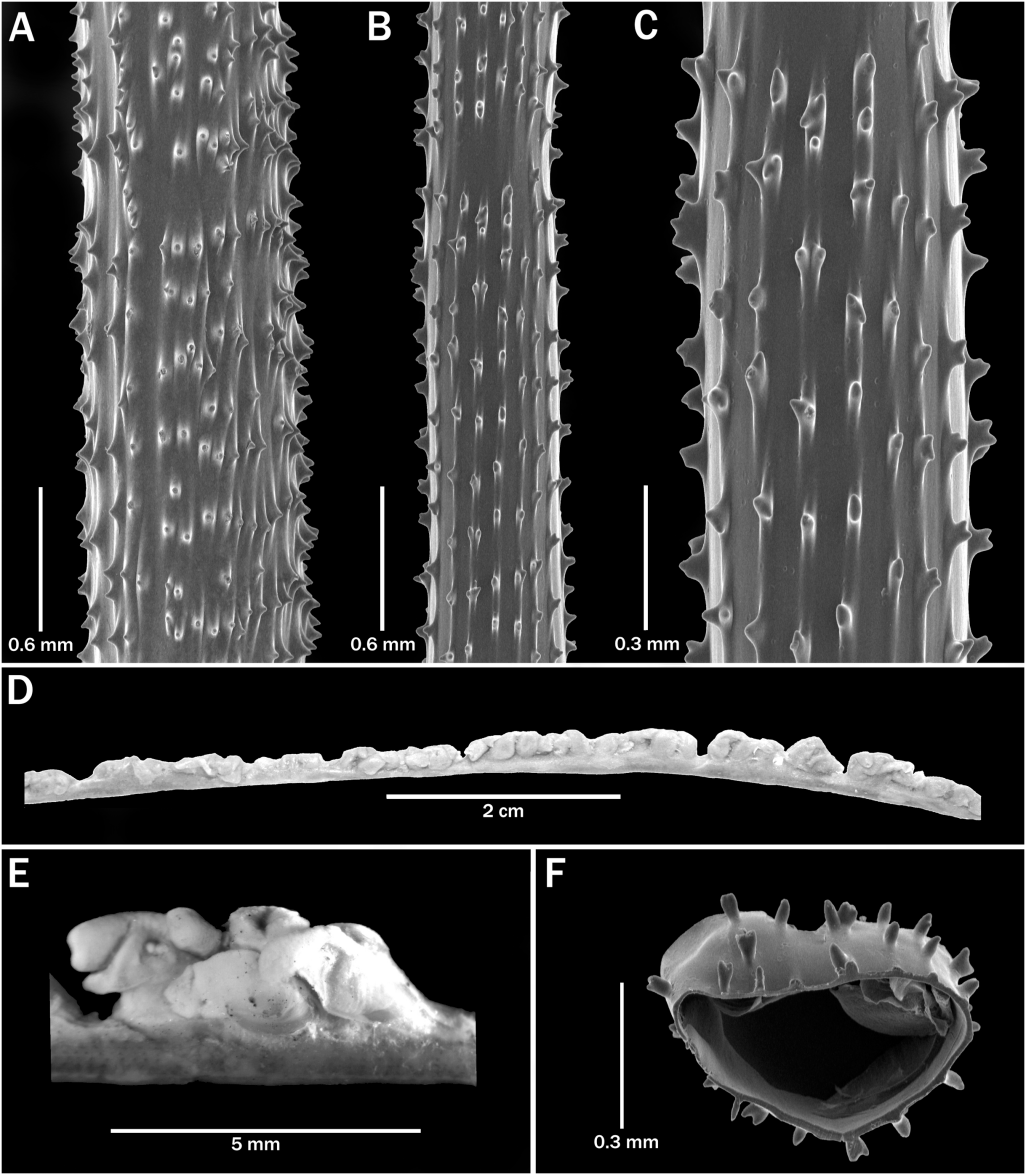
*Stichopathes robusta* Gravier, 1921, holotype, MOM INV-0021221 (Schizopathidae *incertae sedis*). (A–C, F) Sections of pinnules showing size and arrangement of spines. (D) Section of corallum. (E) Polyp.

*Stichopathes robusta* is a problematic nominal species with an unknown colony shape and pinnulation pattern which was originally described from a fragment (Fig. 9) is proposed here above as Schizopathidae *incertae sedis*. It differs from the new species by having more rows of spines on the pinnules (9–13 *vs*. 6–8 visible from side) and very characteristically multilobed and furcated spines (Figs. 9B, 9C and 9F).

**Determinations from underwater imagery.** *Bathypathes pseudoalternata* sp. nov. forms a characteristically *Bathypathes*-type monopodial colony with two rows of long pinnules, longest near the middle of the pinnulated section. Upright colonies of *Bathypathes pseudoalternata* sp. nov. with pinnules of a uniform length and forming an inner angle not exceeding 180° can be easily distinguished from the characteristically windsock-like colonies of representatives of the genus *Alternatipathes* (Supplemental Material S4). Compared to *Alternatipathes, B. pseudoalternata* sp. nov. has an upright corallum with a straight pinnulated part (*vs*. bent pinnulated part in *Alternatipathes* spp.), pinnules of uniform length and density (*vs*. decreasing regularly distally), and a constant distal angle formed by the pinnules and the stem along different parts of the corallum (*vs*. decreasing of distal angle near the top) (Supplemental Material S4). The new species can be distinguished from most of the hitherto known species of the genus *Bathypathes* by its alternating pinnules and from *Bathypathes platycaulus* (normally reported from shallower depths of less than 150 m) by less densely arranged pinnules (16–30 *vs*. > 50 per 10 cm).

**Etymology.** Species name “*pseudoalternata*” derived from species name “*alternata*” with Greek prefix “pseudo”, meaning “false”, is chosen to denote that for many years this species was misidentified as *Bathypathes alternata*.

**Distribution.** Currently recorded from the North Pacific, the North Atlantic, New Guinea, the Great Australian Bight, New Zealand; Tasmanian seamounts, at depths ranging from 331 to 4,152 m (Fig. 7).

**Distribution of black corals in the lower bathyal and abyssal zones**

Based on published data and additional distributional records checked by the authors, of the 285 currently described black coral species, many occur in the deep sea (Table 3). We analyzed lower bathyal and abyssal distributions separately because several species were reported from the transition zone and thus a single record belongs to both abyssal and lower bathyal zones. We did not use GBIF (www.gbif.org) distribution records that were not checked by us due to the high risk of possible misidentifications introduced by machine observations. Thus, several GBIF distributional records for *Alternatipathes alternata* based on machine observation of underwater photographs appeared in fact to be holothurian feces. Also, museum collections data determined earlier, especially before 2001, have to be checked for consistency as after 2001 several taxonomic revisions were published and many new species and genera were described (see *e.g*., Opresko, 2002, 2005, 2019; Molodtsova & Opresko, 2017; Opresko & Wagner, 2020; Opresko & Molodtsova, 2021 for Schizopathidae). We caution others not to use such data without first checking the identifications.

**Table 3:** Species with lower bathyal and abyssal distribution across hitherto known genera of Antipatharia.

Genus	Total number of nominal species*	Nominal species reported in the range 800–3,500 m	Nominal species reported below 3,500 m	All nominal species reported below 800 m**	Potential new species*** reported below 800 m*
ANTIPATHIDAE					
***Allopathes***	3	1	0	1	0
***Antipathes***	69	6	0	6	0
*Blastopathes*	1	0	0	0	0
*Cirrhipathes*	14	0	0	0	0
*Hillopathes*	1	0	0	0	0
*Pseudocirrhipathes*	2	0	0	0	0
*Pteropathes*	2	0	0	0	0
***Stichopathes***	30	6	0	6	1
APHANIPATHIDAE					
*Acanthopathes*	5	0	0	0	0
*Acanthosaropathes*	1	0	0	0	0
*Anozopathes*	2	0	0	0	0
*Aphanipathes*	4	0	0	0	0
***Aphanostichopathes***	4	4	0	4	0
*Asteriopathes*	3	0	0	0	0
*Distichopathes*	3	0	0	0	0
***Elatopathes***	1	1	0	1	0
***Phanopathes***	5	1	0	1	1
*Pteridopathes*	2	0	0	0	0
*Rhipidipathes*	3	0	0	0	0
*Tetrapathes*	2	0	0	0	0
Aphanipathidae *indet*.	0	0	0	0	1
CLADOPATHIDAE					
***Chrysopathes***	5	3	0	3	0
***Cladopathes***	1	1	0	1	0
***Heteropathes***	5	4	1	5	0
*Hexapathes*	4	0	0	0	0
***Sibopathes***	2	2	0	2	0
***Trissopathes***	4	3	0	3	0
LEIOPATHIDAE					
***Leiopathes***	9	7	1	7	0
MYRIOPATHIDAE					
***Antipathella***	5	1	0	1	0
*Cupressopathes*	6	0	0	0	0
*Myriopathes*	11	0	0	0	0
*Plumapathes*	2	0	0	0	0
*Tanacetipathes*	10	0	0	0	0
SCHIZOPATHIDAE					
***Abyssopathes***	3	2	3	3	0
***Alternatipathes***	4	3	2	4	0
***Bathypathes***	13	10	6	13	2
***Dendrobathypathes***	4	4	0	4	2
*Dendropathes*	2	0	0	0	0
***Lillipathes***	4	3	0	3	0
***Parantipathes***	10	7	0	7	1
*Saropathes*	2	0	0	0	0
***Schizopathes***	3	3	1	3	0
***Stauropathes***	4	4	0	4	0
***Taxipathes***	1	1	0	1	0
***Telopathes***	2	2	0	2	1
***Umbellapathes***	3	3	0	3	0
**Schizopathidae *indet***	0	0	0	0	1
STYLOPATHIDAE					
***Tylopathes***	2	0	0	0	1
***Triadopathes***	1	1	0	1	0
***Stylopathes***	6	1	0	1	0
**Total**	**285**	**84**	**14**	**90**	**11**

**Note:**

Genera with all species known from shallower depth are not highlighted. For more data see Supplemental Materials S2 and S3 (*) according to WoRMS accessed 01-11-2021. (**) Counting both lower bathyal and abyssal species. (***) Based on published data.

**Figure 10:**
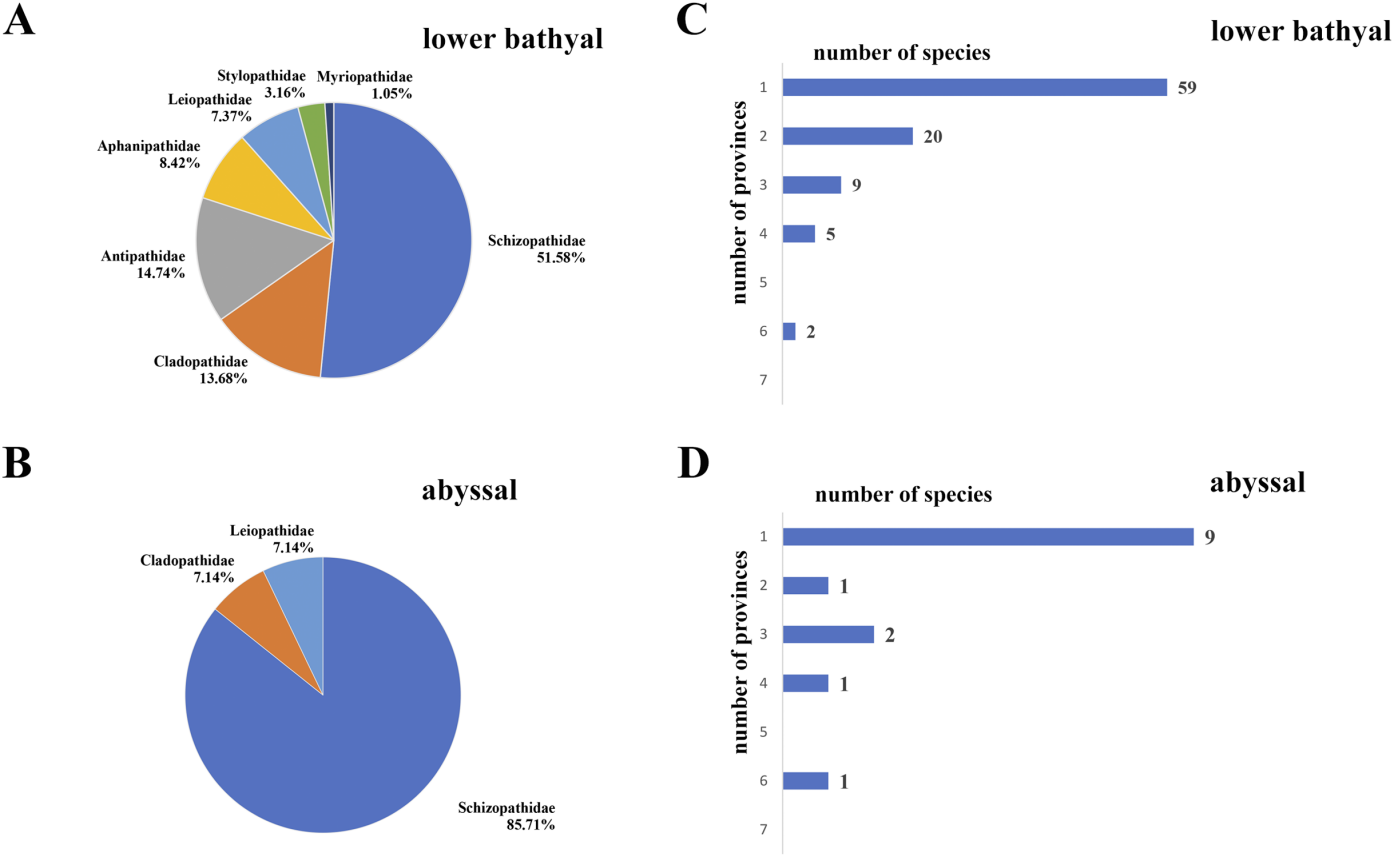
Patterns of distribution of deep-sea antipatharians. Black coral family diversity in (A) lower bathyal zone and (B) abyssal zone. Frequency distribution of black coral species based on number of provinces in which they occur in (C) lower bathyal zone and (D) abyssal zone.

Based on our compilation (Supplemental Material S2, Table 3) 31.57% of the total number of species described (90 nominal species in 27 genera) occur below 800 m, and of this number 14 species in 6 genera occur below 3,500 m. The majority of the lower bathyal and abyssal black corals belong to the family Schizopathidae, representing 51.58% (42 nominal and seven potential new species) of the total number black coral species (95) reported from depths of 800–3,500 m and 85.71% (12 of 14 nominal species) from depths below 3,500 m (Figs. 10A and 10B). Only two schizopathid genera (*Dendropathes* Opresko, 2005 and *Saropathes* Opresko, 2002) are known exclusively at depths less than 800 m. Genera *Dendrobathypathes* Opresko, 2002, *Parantipathes* Brook, 1889 and *Stauropathes* Opresko, 2002 are predominately bathyal with the majority of species known from lower bathyal depths. Genus *Abyssopathes* Opresko, 2002 is known mainly from abyssal depths (Molodtsova & Opresko, 2017), whereas genera *Alternatipathes, Bathypathes*, and *Schizopathes* are equally present in the lower bathyal and abyssal zones. Six of 14 abyssal species are *Bathypathes* species (Table 3).

Based on existing records (Supplemental Material S2), the most diverse lower bathyal province, in terms of number of species reported, is the North Atlantic (BY4) with 43 species reported, followed by New Zealand-Kermadecs (BY6:18 species), Indian Ocean (BY11:16 species), Subantarctic (BY10) and South Atlantic (BY13), each with 15 species reported. Not a single species of black coral has been recorded from the High Arctic (BY1) or from the Southeast Pacific Ridges (BY5) lower bathyal provinces. In the abyssal zone, the most diverse province is the North Pacific (AB13) with 8 species reported below 3,500 m, followed by the Indian (AB8:5 species); and North Atlantic (AB2), Antarctica East (AB6) and Equatorial Pacific (AB11), each with four reported species. The great majority (59 species of 95 determined to the species level including potential new species for the lower bathyal and 9 species of 14 determined to the species level for the abyssal zone) occur in a single biogeographical province (Figs. 10C and 10D). Twenty lower bathyal and one abyssal species have been reported from only two biogeographic provinces and 20 species are known from three and more provinces. *Bathypathes pseudoalternata* sp. nov. described here is reported from six lower bathyal provinces, one abyssal province (see Supplemental Material S2), and also from mid-bathyal depths of the Gulf of Mexico, North-West and North-East Atlantic, slopes of Hawaii, New Zealand and Madagascar.

## Discussion

Although many species inhabiting the slopes of oceanic ridges and seamounts have been reported to show a high degree of endemism (see *e.g*. Parin, Mironov & Nesis, 1997; Richer de Forges, Koslow & Poore, 2000); seamount-scale endemism has not yet been shown to occur in antipatharians, although it should be noted that only one study using a limited number of mitochondrial markers has evaluated this possibility (Thoma et al., 2009). As it is possible to see from existing distribution data (Supplemental Material S2) most of the hitherto known species of black corals are limited to a single biogeographical province or two adjacent provinces. Only a few species demonstrate a wider geographic distribution pattern, including the new species, *Bathypathes pseudoalternata*, described here. On the other hand, it is possible to see that data on the distribution of the deep-sea black corals are critically scarce and uneven. Many species are known from a single locality. Consequently, some identified trends reflect sampling effort rather than actual distribution patterns. Many new species are currently being described, but there is a crucial need for local faunal reviews and faunal lists, to document the vertical and geographical distribution patterns. Given the high sampling effort in the High Arctic province (BY1), the absence of antipatharian records from this region likely is due to black corals being rare in this region. In contrast, the fact that only a single species is recorded from the Southeast Pacific Ridges lower bathyal province (BY5) is likely due to limited sampling efforts in this region. Abyssal provinces are even less studied in term of black corals (see Supplemental Material S2). Most local faunal reviews consider shallow-water species only, with a few exceptions, including the: North Atlantic (Molodtsova, 2006; Braga-Henriques et al., 2013; Molodtsova, 2014), South Atlantic (Lima, Cordeiro & Perez, 2019), Great Australian Bay (MacIntosh et al., 2018), South Pacific (Horowitz, Opresko & Bridge, 2018) and Central Pacific (Molodtsova & Opresko, 2017) with a few additional reviews forthcoming in the near future. The material of *Bathypathes pseudoalternata* sp. nov. examined as part of this study came from six lower bathyal provinces (North Atlantic BY4, New Zealand-Kermadecs BY6, Subantarctic BY10, Indian Ocean BY11, West Pacific B12, and North Pacific BY14), one abyssal province (North Atlantic AB2), and also from mid-bathyal depths of the Gulf of Mexico, North-West and North-East Atlantic, slopes of Hawaii, New Zealand and Madagascar. Consequently, this species has a cosmopolitan distribution.

While our study focused on morphological analyses, there also exists genetic support for such a wide distribution pattern of *Bathypathes pseudoalternata*. Brugler, Opresko & France (2013) sequenced ten specimens of *B. pseudoalternata* (identified at the time as *B. alternata*). Nine of these specimens were collected in the North Atlantic and one was collected in the North Pacific near Hawaii (paratype, BPBM D1775). Brugler, Opresko & France (2013: Table S1) found that all 10 specimens share identical haplotypes for three mitochondrial DNA regions (*igrW* between *trnW-nad2*; *igrN* between *nad5-nad1*; and *cox3* to *cox1*, excluding the *Igr*) which were different from all hitherto studied species in the genus *Bathypathes*. Subsequent morphological study of some of the same specimens sequenced by Brugler, Opresko & France (2013) has shown that a unique combination of haplotypes across all 3 mt gene regions can be indicative of a genus level taxon (see description of *Telopathes magna* MacIsaac & Best, 2013 in MacIsaac et al., 2013) and a unique combination of haplotypes across 2 or 3 mt gene regions can be indicative of species-level taxa (see descriptions of *Bathypathes alaskensis* and *B. ptiloides* in Opresko & Molodtsova, 2021). Based on these results we postulate that the small morphological differences seen among some *B. pseudoalternata* sp. nov. specimens (particularly in the shape of the spines) in the absence of genetic differences, represents only intraspecific variation.

In a more recent phylogenetic study, Bilewitch & Tracey (2020) sequenced three mitochondrial gene regions and two nuclear gene regions for a number of *Bathypathes* specimens collected from the seas around New Zealand. The mt gene regions were *16S*, *TrnW-igr-ND2* and *ND5-igr-ND1*. One of the specimens sequenced by Bilewitch & Tracey (2020) was identified as *Bathypathes alternata* (NIWA 64561); however, examination of photos of the specimen (provided by J. Horowitz) indicate that the gross morphology of the colony matches the morphotype of *B*. *pseudoalternata*. In their phylogenetic reconstructions using *TrnW-igr-ND2* and *ND5-igr-ND1*, Bilewitch & Tracey (2020) included the sequence data for North Atlantic specimens of *Bathypathes* “*alternata”* (= *B*. *pseudoalternata*) from the Brugler, Opresko & France (2013) study. The resulting phylogenetic reconstruction using *TrnW-igr-ND2* revealed that the New Zealand specimen had the same haplotype as the four North Atlantic specimens. In the phylogenetic reconstruction using *ND5-igr-ND1* (Bilewitch & Tracey, 2020, Appendix Table B2), NIWA 64561 was indistinguishable from six North Atlantic specimens of *B*. *pseudoalternata* sp. nov., one of which was YPM IZ 028566 (field number MAN 201-1), the same specimen that Chery et al. (2018) showed had a *nad5-IGR-nad1* haplotype identical to that of three Hawaiian specimens of *B*. *pseudoalternata*. In both of the phylogenetic reconstructions of Bilewitch & Tracey (2020), the New Zealand and North Atlantic specimens of *B*. *pseudoalternata* sp. nov. formed a separate subclade that fell within a larger clade containing subclades of specimens of *Stauropathes*, *Bathypathes*, and *Telopathes* MacIsaac & Best, 2013 in MacIsaac et al., 2013 as well as two subclades that the authors considered unknown genera.

It should be noted, however, that depending on the methodologies employed, phylogenetic analyses using a limited number of mitochondrial regions may not always be adequate to distinguish between closely related species. For example, in their analysis, and using *TrnW-igr-ND2* data taken from GenBank, Bilewitch & Tracey (2020) did not find any differences between *B*. *alaskensis* Opresko & Molodtsova, 2021 (KF054475), *B*. *ptiloides* Opresko & Molodtsova, 2021 (KF054479), and numerous New Zealand specimens identified as *B*. *patula*. However, in Brugler, Opresko & France (2013), the holotype of *B*. *ptiloides* was found to have different haplotypes from *B*. *alaskensis* across 3 mt gene regions, *IgrW*, *IgrN*, and *cox3-cox1* (none of the New Zealand specimens identified as *B*. *patula* in Bilewitch & Tracey were sequenced by Brugler, Opresko & France (2013)). In some cases, limited mitochondrial gene data are even insufficient to separate three different genera in the family Schizopathidae (see Brugler, Opresko & France, 2013 regarding the ‘trigeneric complex’).

Although the available genetic studies provide supporting data that *B*. *pseudoalternata* is cosmopolitan, the absence of genetic differences in a limited number of mt gene regions in geographically separate populations does not preclude the possibility that new genetic data will reveal differences in these populations; therefore, our conclusions are largely based on the available morphological evidence. The situation may change with development of new genetic approaches and the use of different mitochondrial and nuclear markers. Therefore, the geographical variations described here, particularly in the morphology of the spines, may eventually prove to be indicative of separate species. However, for the time being there is no reliable morphological or molecular evidence to distinguish between specimens from the Pacific and Atlantic oceans and New Zealand described here as *Bathypathes pseudoalternata*. Similar results showing a wide geographic distribution based on both morphological and molecular analyses was recently reported for the shallow and mesophotic antipatharian species *Antipathes grandis* Verrill, 1922 in the family Antipathidae (Gress et al., 2020).

It is noteworthy that many of the records of *Bathypathes pseudoalternata* sp. nov. identified as part of this study (Fig. 7) are from seafloor areas that are known to contain high concentrations of commercially-valuable seabed minerals, specifically cobalt-rich ferromanganese crusts in the Central Pacific and North Atlantic (see Clark et al., 2011; Miller et al., 2018). This has important implications for the international seabed mining regulations that are currently being developed (Miller et al., 2018). While the growth rate and lifespan of *B. pseudoalternata* has not been studied to date, black corals include some of the slowest growing and longest living organisms on Earth, with lifespans of individual species ranging from centuries to millennia (reviewed in Wagner, Luck & Toonen, 2012). Given the reported slow growth rates that are characteristic for black corals and recognizing the ecological importance of these species, areas inhabited by them should be avoided by extractive industries, including seabed mining activities.

## Conclusions

The new species *Bathypathes pseudoalternata*, common at mid- and lower bathyal depths of the Pacific, Atlantic and Indian oceans, and previously misidentified with the abyssal species *Alternatipathes alternata* (Brook, 1889), is formally described based on morphological data. The new species is virtually cosmopolitan and has been reported from continental slopes, ridges and seamounts, including areas covered by cobalt-rich ferromanganese crusts at lower bathyal depths in six deep-sea provinces (Watling et al., 2013) in the Pacific, Atlantic and Indian oceans. Available genetic (MacIsaac et al., 2013; Brugler, Opresko & France, 2013; Chery et al., 2018; Bilewitch & Tracey, 2020) and morphological data (this study) are not sufficient to distinguish between geographically distant populations, therefore leading to the hypothesis that these geographically dispersed populations all belong to the same species. We demonstrated a critical need for local faunal reviews of deep-sea black corals. Existing data on the distribution of the deep-sea black corals are critically scarce, uneven and reflect sampling effort, thereby masking actual distribution patterns. Also, we warn that the identifications of corals in underwater photographs by machine learning need to be checked by experts before being used as distribution records. Finally, the finding of a new species of black coral, which creates habitat for various other associated species and is likely slow growing, indicates that areas where it occurs should be avoided by extractive industries, particularly seabed mining.

## Supplemental Information

10.7717/peerj.12638/supp-1 Supplemental Information 1 *Bathypathes pseudoalternata* sp. nov. photo records examined as part of this study. Only photographs of colonies that matched the external diagnostic features in overall colony morphology and branching pattern were included in the analysis. Catalogue numbers from the NOAA National Database of Deep-Sea Corals and Sponges (Database) or the NOAA Ocean Exploration Benthic Deepwater Animal Identification Guide (Guide). * collected

10.7717/peerj.12638/supp-2 Supplemental Information 2 Bathymetric and geographical distribution of Antipatharia reported from lower bathyal (801–3,500 m) and abyssal (3,501–6,500 m) zones. (A) Species list and distribution. Species of uncertain taxonomic position not included in analysis highlighted in red. Potential new species highlighted in blue. Doubtful records in parentheses. Regions *sensu* Watling et al. (2013). (B) References.

10.7717/peerj.12638/supp-3 Supplemental Information 3 Additional species distribution records based on specimens identified/checked by T.N. Molodtsova, D.M. Opresko. Catalog numbers and locations of unpublished records used in Supplementary Material S2

10.7717/peerj.12638/supp-4 Supplemental Information 4 Comparison of underwater imagery of *Bathypathes pseudoalternata* sp. nov. and *Alternatipathes* spp. links to underwater images and video showing distinctive gross morphology and associated fauna

## List of abbreviations and acronyms

BPBM: Bernice P. Bishop Museum
CSIRO: Commonwealth Scientific and Industrial Research Organization
DSRV: Deep Submergence Research Vehicle
FV: Fishing vessel
FRV: Fishery Research vessel
HOV: Human Occupied Vehicle
IORAS: P.P. Shirshov Institute of Oceanology of the Russian Academy of Sciences
MNHN: Muséum national d’Histoire naturelle
MOM: Musée Océanographique de Monaco
NIWA: National Institute of Water and Atmospheric Research (formerly NZOI)
NMNH: National Museum of Natural History, Smithsonian Institution
NOAA: National Oceanographic and Atmospheric Administration
NOCS: National Oceanography Centre
NZOI: New Zealand Oceanographic Institute
ROV: Remotely operated vehicle
RV: Research vessel
SEM: Scanning Electron Microscope
TMAG: Tasmanian Museum
UKNHM: Natural History Museum, London,
USNM: Prefix for catalogue numbers of specimens at the NMNH
YPM IZ: Yale Peabody Museum, Invertebrate Zoology

## Additional Information and Declarations

### Data Availability

The following information was supplied regarding data availability: The accession numbers and location for each specimen studied are available in Table 1 and here: BPBM D1775 (paratype)-the Bernice P. Bishop Museum (BPBM) in Honolulu, HI, USA; Ifremer Brest reference collection no number-Institut de recherche intégré en sciences marines in Plouzane, France; IORAS CNI00015-the P.P. Shirshov Institute of Oceanology (IORAS) in Moscow, Russia; MNHN IK-2012-12009, MNHN IK-2012-12064, MNHN IK-2012-12124, MNHN IK-2012-12174, MNHN IK-2014-217-the Muséum national d’Histoire naturelle (MNHN) in Paris, France; MOM INV-0021212-the Musée Océanographique de Monaco (MOM), Monaco; NIWA 24195, NIWA 4299, NIWA 4301, NIWA 4302-the National Institute of Water and Atmospheric Research (NIWA) in Wellington, New Zealand; NOCS 9015-the Discovery Collection of the National Oceanography Centre (NOC) in Southampton, UK; TMAG K1355, TMAG K4515-the Tasmanian Museum and Art Gallery (TMAG) in Hobart, Tasmania, Australia; USNM 1071041, USNM 1071043, USNM 1071044 (Holotype), USNM 1071412, USNM 1093063, USNM 1139376, USNM 1139377, USNM 1163565, USNM 1163568, USNM 1163569, USNM 1163570, USNM 1163571, USNM 1174701, USNM 77110, USNM 83547, USNM 96356, USNM 98843, YPM IZ 028567 the National Museum of Natural History (NMNH), Smithsonian Institution in Washington, DC, USA.

### New Species Registration

The following information was supplied regarding the registration of a newly described species: Publication LSID: urn:lsid:zoobank.org:pub:58FF4531-1478-4462-84ED-A7E96920710E. *Bathypathes pseudoalternata* sp. nov. Species name: LSID: urn:lsid:zoobank.org:act:DE0DC39A-4A11-4AC1-9EB1-F632FE9E3D1F.

## References

[ref-1] Barnich R, Beuck L, Freiwald A (2013). Scale worms (Polychaeta: Aphroditiformia) associated with cold-water corals in the eastern Gulf of Mexico. Journal of the Marine Biological Association of the United Kingdom.

[ref-2] Berntson EA, France SC, Mullineaux LS (1999). Phylogenetic relationships within the class Anthozoa (phylum Cnidaria) based on nuclear 18S rDNA sequences. Molecular Phylogenetics and Evolution.

[ref-3] Braga-Henriques A, Porteiro FM, Ribeiro PA, de Matos V, Sampaio I, Ocaña O, Santos RS (2013). Diversity, distribution and spatial structure of the cold-water coral fauna of the Azores (NE Atlantic). Biogeosciences.

[ref-4] Bilewitch JP, Tracey D (2020). Protected coral connectivity in New Zealand. Final Report prepared by NIWA for the Conservation Services Programme, Department of Conservation. DOC19306-POP201806. NIWA Client Report 2020222WN: 32. https://www.doc.govt.nz/globalassets/documents/conservation/marine-and-coastal/marine-conservation-services/reports/final-reports/pop2018-06-protected-coral-connectivity-final-report.pdf.

[ref-5] Brook G (1889). Report on the Antipatharia collected by H, Challenger during the years 1873–1876. Report on the Scientific Results of the Voyage of H.M.S. Challenger Zoology.

[ref-6] Brugler MR, Opresko DM, France SC (2013). The evolutionary history of the order Antipatharia (Cnidaria: Anthozoa: Hexacorallia) as inferred from mitochondrial and nuclear DNA: implications for black coral taxonomy and systematics. Zoological Journal of the Linnean Society.

[ref-7] Cairns SD, Opresko DM, Hopkins TS, Schroeder WW (1993). New records of deep-water Cnidaria (Scleractinia & Antipatharia) from the Gulf of Mexico. Gulf of Mexico Science.

[ref-8] Cantwell K, Pomponi S, Fryer P (2019). Oceanographic data and information collected during the EX1605L3 (CAPSTONE CNMI & Mariana Trench MNM (ROV & Mapping)) expedition on NOAA Ship OKEANOS EXPLORER in the North Pacific Ocean from 2016-06-17 to 2016-07-10 (NCEI Accession 0156334). Imagery. NOAA National Centers for Environmental Information. Dataset.

[ref-9] Cantwell K, Chaytor J, Galvez K, Mizell K, Waller R (2021). Video and imagery data collected during the EX2104 2021 North Atlantic Stepping Stones (ROV & Mapping) expedition on NOAA Ship OKEANOS EXPLORER in the North Atlantic Ocean from 2021-06-30 to 2021-07-29 (NCEI Accession 0240591). Imagery. NOAA National Centers for Environmental Information. Dataset. https://doi.org/10.7289/v5c24tgx.

[ref-10] Chery N, Parra K, Stein D, Distel D, Appiah-Madson H, Ross R, Sanon E, Alomari N, Johnson R, Vasovic A, Horowitz A, Popa H, Short B, Kourehjan D, Vasquez DM, Rodriguez E, Opresko DM, Brugler MR (2018). Partnering with the Ocean Genome Legacy to advance our understanding of black corals (Order Antipatharia). Poster presentation. 15th Deep-Sea Biology Symposium, September 9–14, 2018. Monterey, CA. https://2328be37-4b2b-4774-894d-916f74520ece.filesusr.com/ugd/5fcd02_e4a81ea0a872469196f20467f9f9ee4c.pdf.

[ref-11] Clark MR, Kelley C, Baco A, Rowden A (2011). Fauna of cobalt-rich ferromanganese crust seamounts.

[ref-12] De Clippele LH, Huvenne VAI, Molodtsova TN, Roberts JM (2019). The diversity and ecological role of non-scleractinian corals (Antipatharia and Alcyonacea) on scleractinian cold-water coral mounds. Frontiers in Marine Science.

[ref-13] Etnoyer PJ, Shuler A, Cairns SD (2020). Deep-Sea Coral Taxa in the U.S. Gulf of Mexico: depth and geographical distribution (v. 2020). https://deepseacoraldata.noaa.gov/NOAA_DSC-Species-List_GulfofMexico_Etnoyer-etal_2020.pdf.

[ref-14] Gravier CJ (1921). Antipathaires provenant des campagnes des yachts *Princesse-Alice* et *Hirondelle II* (1903–1913). Résultats des Campagnes Scientifiques du Prince de Monaco.

[ref-15] Gress E, Opresko DM, Brugler MR, Wagner D, Eeckhaut I, Terrana L (2020). Widest geographic distribution of a shallow and mesophotic antipatharian coral (Anthozoa: Hexacorallia): *Antipathes grandis* Verrill, 1928-confirmed by morphometric and molecular analyses. Marine Biodiversity Records.

[ref-16] Horowitz J, Opresko DM, Bridge TCL (2018). Black corals (Anthozoa: Antipatharia) from the deep (916 m-2542 m) Coral Sea, north-eastern Australia. Zootaxa.

[ref-17] Horowitz J, Brugler MR, Bridge TC, Cowman PF (2020). Morphological and molecular description of a new genus and species of black coral (Cnidaria: Anthozoa: Hexacorallia: Antipatharia: Antipathidae: *Blastopathes*) from Papua New Guinea. Zootaxa.

[ref-18] Hourigan TF, Etnoyer PJ, McGuinn RP, Whitmire CE, Dorfman DS, Dornback M, Cross SL, Sallis DE (2015). An introduction to NOAA’s national database for deep-sea corals and sponges.

[ref-19] Hourigan TF, Cairns SD, Reed JK, Ross SW (2020). Deep-sea coral taxa in the U.S. Southeast region: depth and geographical distribution (v. 2020). https://deepseacoraldatanoaagov/library/2020-regionaldeep-sea-coral-species-list.

[ref-20] Howell KL, Davies JS, Allcock AL, Braga-Henriques A, Buhl-Mortensen P, Carreiro-Silva M, Dominguez-Carrió C, Durden JM, Foster NL, Game CA (2019). A framework for the development of a global standardised marine taxon reference image database (SMarTaR-ID) to support image-based analyses. PLOS ONE.

[ref-21] Kennedy B, Demopoulos A, Auscavitch S (2017). Oceanographic data and information collected during the EX1703 Howland/Baker PRIMNM and PIPA (ROV/Mapping) expedition on NOAA Ship OKEANOS EXPLORER in the South Pacific Ocean from 2017-03-07 to 2017-03-29 (NCEI Accession 0162616). NOAA National Centers for Environmental Information. Dataset. https://doi.org/10.7289/v5sj1hw0.

[ref-22] Lapointe AE, Watling L, France SC, Auster PJ (2020). Megabenthic assemblages in the lower bathyal (700–3000 m) on the New England and Corner Rise seamounts, Northwest Atlantic. Deep Sea Research Part I: Oceanographic Research Papers.

[ref-23] Lima MM, Cordeiro RTS, Perez CD (2019). Black Corals (Anthozoa: Antipatharia) from the Southwestern Atlantic. Zootaxa.

[ref-24] MacIntosh H, Althaus F, Williams A, Tanner JE, Alderslade P, Ahyong ST, Bax N, Criscione F, Crowther AL, Farrelly CA, Finn JK, Goudie L, Gowlett-Holmes K, Hosie AM, Kupriyanova E, Mah C, McCallum AW, Merrin KL, Miskelly A, Mitchell ML, Molodtsova T, Murray A, O’Hara TD, O’Loughlin PM, Paxton H, Reid AL, Sorokin SJ, Staples D, Walker-Smith G, Whitfield E, Wilson RS (2018). Invertebrate diversity in the deep Great Australian Bight (200–5000 m). Marine Biodiversity Records.

[ref-25] MacIsaac KG, Best M, Brugler MR, Kenchington EL, Anstey LJ, Jordan T (2013). *Telopathes magna* gen. nov., spec. nov. (Cnidaria: Anthozoa: Antipatharia: Schizopathidae) from deep waters off Atlantic Canada and the first molecular phylogeny of the deep-sea family Schizopathidae. Zootaxa.

[ref-26] Miller KA, Thompson KF, Johnston P, Santillo D (2018). An overview of seabed mining including the current state of development, environmental impacts, and knowledge gaps. Frontiers of Marine Science.

[ref-48] Molodtsova TN, Mironov AN, Gebruk AV, Southward AJ (2006). Black corals (Antipatharia: Anthozoa: Cnidaria) of North-East Atlantic. Biogeography of the North Atlantic seamounts.

[ref-27] Molodtsova TN (2014). Deep-sea fauna of European seas: an annotated species check-list of benthic invertebrates living deeper than 2000 m in the seas bordering Europe, Antipatharia. Invertebrate Biology.

[ref-28] Molodtsova TN, Britayev TA, Martin D, Goffredo S, Dubinsky Z (2016). Cnidarians and their polychaete symbionts. The Cnidaria, Past, Present and Future.

[ref-29] Molodtsova TN, Opresko DM (2017). Black corals (Anthozoa: Antipatharia) of the Clarion-Clipperton Fracture Zone. Marine Biodiversity.

[ref-30] Opresko DM (1974). A study of the classification of the Antipatharia (Coelenterata: Anthozoa) with redescriptions of eleven species.

[ref-31] Opresko DM (2002). Revision of the Antipatharia (Cnidaria: Anthozoa). Part II. Schizopathidae. Zoologische Mededelingen Leiden.

[ref-32] Opresko DM (2005). New genera and species of antipatharian corals (Cnidaria: Anthozoa) from the North Pacific. Zoologische Mededelingen Leiden.

[ref-33] Opresko DM, Felder DF, Camp DK (2009). Antipatharia (Cnidaria) of the Gulf of Mexico. Gulf of Mexico Origin, Waters, and Biota: Biota Biodiversity.

[ref-34] Opresko DM (2019). New species of black corals (Cnidaria: Anthozoa: Antipatharia) from the New Zealand region, part 2. New Zealand Journal of Zoology.

[ref-35] Opresko DM, Tracey D, Mackay E (2014). Antipatharia (black corals) for the New Zealand region. A field guide of commonly sampled New Zealand black corals including illustrations highlighting technical terms and black coral morphology.

[ref-36] Opresko DM, Wagner D (2020). New species of black corals (Cnidaria: Anthozoa: Antipatharia) from deep-sea seamounts and ridges in the North Pacific. Zootaxa.

[ref-37] Opresko DM, Molodtsova TN (2021). New species of deep-sea antipatharians from the North Pacific (Cnidaria: Anthozoa: Antipatharia), Part 2. Zootaxa.

[ref-38] Parin NV, Mironov AN, Nesis KN (1997). Biology of the Nazca and Sala y Gomez submarine ridges, an outpost of the Indo-West Pacific fauna in the eastern Pacific Ocean: composition and distribution of the fauna, its communities and history. Advances in Marine Biology.

[ref-39] Parrish FA, Baco-Taylor AR, Kelley CD, Cairns SD, Hourigan TF (2020). Deep-sea coral taxa in the Hawaiian Archipelago and Johnston Atoll depth and geographical distribution (v 2020). https://deepseacoraldata.noaa.gov/NOAA_DSC-Species-List_Hawaii_Parrish-etal_2020.pdf.

[ref-40] Pasternak FA (1977). Antipatharia. Galathea Report.

[ref-41] Richer de Forges B, Koslow JA, Poore GCB (2000). Diversity and endemism of the benthic seamount fauna in the southwest Pacific. Nature.

[ref-42] Thoma JN, Pante E, Brugler MR, France SC (2009). Deep-sea octocorals and antipatharians show no evidence of seamount-scale endemism in the NW Atlantic. Marine Ecology Progress Series.

[ref-43] Totton AK (1923). Coelenterata. Part III. Antipatharia (and their cirripede commensals). British Antarctic (Terra Nova) Expedition, 1910, natural history report. Zoology.

[ref-44] Wagner D, Waller RG, Toonen RJ (2011). Sexual reproduction of Hawaiian black corals, with a review of the reproduction of antipatharians (Cnidaria: Anthozoa: Hexacorallia). Invertebrate Biology.

[ref-45] Wagner D, Luck D, Toonen RJ (2012). The biology and ecology of antipatharians (Cnidaria: Anthozoa: Hexacorallia). Advances in Marine Biology.

[ref-46] Watling L, Guinotte J, Clark MR, Smith CR (2013). A proposed biogeography of the deep ocean floor. Progress in Oceanography.

[ref-47] Yesson C, Bedford F, Rogers AD, Taylor ML (2017). The global distribution of deep-water Antipatharia habitat. Deep Sea Research Part II: Topical Studies in Oceanography.

